# Small molecule MarR modulators potentiate metronidazole antibiotic activity in aerobic *E. coli* by inducing activation by the nitroreductase NfsA

**DOI:** 10.1016/j.jbc.2024.107431

**Published:** 2024-05-31

**Authors:** Thibault Caradec, Coline Plé, Giuseppe Sicoli, Ravil Petrov, Elizabeth Pradel, Cécilia Sobieski, Rudy Antoine, Maylis Orio, Adrien Herledan, Nicolas Willand, Ruben Christiaan Hartkoorn

**Affiliations:** 1 Univ. Lille, CNRS, Inserm, CHU Lille, Institut Pasteur Lille, U1019 - UMR 9017 - CIIL - Center for Infection and Immunity of Lille, Lille, France; 2 CNRS UMR 8516, Univ. Lille, LASIRE – Laboratory of Advanced Spectroscopy on Interactions, Reactivity and Environment, Villeneuve d’Ascq, France; 3 Aix Marseille Univ., CNRS, Centrale Marseille, iSm2, Marseille, France; 4 Univ. Lille, Inserm, Institut Pasteur de Lille, U1177 - Drugs and Molecules for Living Systems, Lille, France

**Keywords:** antibiotic action, enzyme mechanism, antibiotic, *E. coli*, transcription regulation, nitroreductase, drug action, radical, metronidazole, boosting

## Abstract

Antibiotic-resistant *Enterobacterales* pose a major threat to healthcare systems worldwide, necessitating the development of novel strategies to fight such hard-to-kill bacteria. One potential approach is to develop molecules that force bacteria to hyper-activate prodrug antibiotics, thus rendering them more effective. In the present work, we aimed to obtain proof-of-concept data to support that small molecules targeting transcriptional regulators can potentiate the antibiotic activity of the prodrug metronidazole (MTZ) against *Escherichia coli* under aerobic conditions. By screening a chemical library of small molecules, a series of structurally related molecules were identified that had little inherent antibiotic activity but showed substantial activity in combination with ineffective concentrations of MTZ. Transcriptome analyses, functional genetics, thermal shift assays, and electrophoretic mobility shift assays were then used to demonstrate that these MTZ boosters target the transcriptional repressor MarR, resulting in the upregulation of the *marRAB* operon and its downstream MarA regulon. The associated upregulation of the flavin-containing nitroreductase, NfsA, was then shown to be critical for the booster-mediated potentiation of MTZ antibiotic activity. Transcriptomic studies, biochemical assays, and electron paramagnetic resonance measurements were then used to show that under aerobic conditions, NfsA catalyzed 1-electron reduction of MTZ to the MTZ radical anion which in turn induced lethal DNA damage in *E. coli*. This work reports the first example of prodrug boosting in *Enterobacterales* by transcriptional modulators and highlights that MTZ antibiotic activity can be chemically induced under anaerobic growth conditions.

*Enterobacterales* represent the leading cause of mortality associated with antimicrobial resistance (1) and an increasing global healthcare burden. The development of novel therapeutic strategies are required to combat multidrug-resistant *Escherichia coli* and *Klebsiella pneumoniae* classed as a critical priority by the World Health Organization (2). A promising strategy that has been pioneered in the fight against *Mycobacterium tuberculosis*, but reasonably unexplored for *Enterobacterales*, is the use of nonantibiotic small molecules to potentiate the bioactivation and antimicrobial activity of prodrug antibiotics (3, 4, 5, 6, 7). In this approach, small synthetic molecules were developed that interfere with *M. tuberculosis* transcriptional regulators, resulting in the overexpression of specific enzymes that bioactivate the prodrug antibiotic ethionamide to its active species. Since the discovery of boosters that induced the known ethionamide bioactivation pathways in *M.* tuberculosis (5), follow-up research also identified transcriptional modulator molecules that awakened alternative “dormant” ethionamide bioactivation enzymes, allowing to overcome current mechanism of clinical ethionamide resistance (4, 7). To date, optimized lead molecules from this drug development program are currently in Phase II of clinical development.

Few prodrug antibiotics have been developed for the treatment of non-*M. tuberculosis* bacterial infections. Among them are nitroaromatic antibiotics such as metronidazole (MTZ), a small nitro-imidazole prodrug with broad-spectrum anti-infective activity on numerous anaerobic or microaerophilic parasites (such as *Giardia lamblia* and *Entamoeba histolytica*) and bacteria (such as *Bacteroides fragilis, Clostridium difficile,* and *Helicobacter pylori*) (reviewed in (8)). In all these organisms, enzymatic nitroreduction plays a pivotal role in activating MTZ to intermediate metabolites with a broad range of intracellular targets. The precise factors that control formation of these reactive MTZ intermediates are currently however debated, and the process seems to be dependent in part on the micro-organism in question. One proposed mechanism of MTZ activation takes place *via* a 1-electron reduction to form MTZ radial anions that react nonspecifically throughout the cell, in particular causing DNA damage. Such 1-electron transfer is believed to be mediated by the ubiquitous pyruvate:ferredoxin oxidoreductase (PFOR) (9) that readily generates MTZ radical anions under microaerophilic/anaerobic conditions (10, 11, 12). Under aerobic conditions, the formation of MTZ radical anions is considered “futile” as oxygen can re-oxidize MTZ radical anions to MTZ, forming superoxide anions in the process (13). However, it remains unclear why this increase in superoxide production in itself is not associated with antibiotic activity in aerobic conditions. An additional described mechanism of MTZ bioactivation is through flavin oxidoreductase-mediated 2- or 4-electron reduction of MTZ to reactive nitroso or hydroxylamine intermediates that can react with nucleophilic moieties in the cells such as DNA bases or amino acids of proteins. Few flavin containing oxidoreductases are known to reduce MTZ as they have a low midpoint redox potential (−486 mV) (14). Nonetheless, flavodoxins in *Trichomonas vaginalis*, *E. histolytica*, *G. lamblia,* and *H. pylori* (15) are capable of 2-electron nitroreduction and are believed to be instrumental in MTZ bioactivation in microaerophilic and anaerobic conditions. Surprisingly, MTZ is ineffective as a prodrug antibiotic against *E. coli* under both anaerobic and aerobic conditions, though it does show antibiotic activity in anaerobic conditions on *E. coli* with impaired DNA-repair systems (16).

Considering the broad range of mechanisms able to mediate MTZ activation, we hypothesized that even in MTZ-resistant bacteria such as *E. coli,* there may be dormant or poorly expressed enzymes which upon induction could boost/potentiate MTZ bioactivation and generate antibiotic activity. In addition, it was considered interesting to investigate if such enzymes could induce MTZ activity under aerobic conditions, which would broaden the therapeutic scope of this approach. To identify potential pathways of MTZ activation in *E. coli* under aerobic conditions, a library of small molecules was screened for their capacity to synergize with MTZ. We identified two related hits and then used genetic, biochemical, and biophysical approaches to characterize how these boosters and a more potent derivative were able to induce MTZ activation and activity in aerobic conditions, revealing novel insight into MTZ-mediated antibiotic activity.

## Results

### Identification of molecules that potentiate MTZ activity in E. coli under aerobic conditions

**Figure 1:**
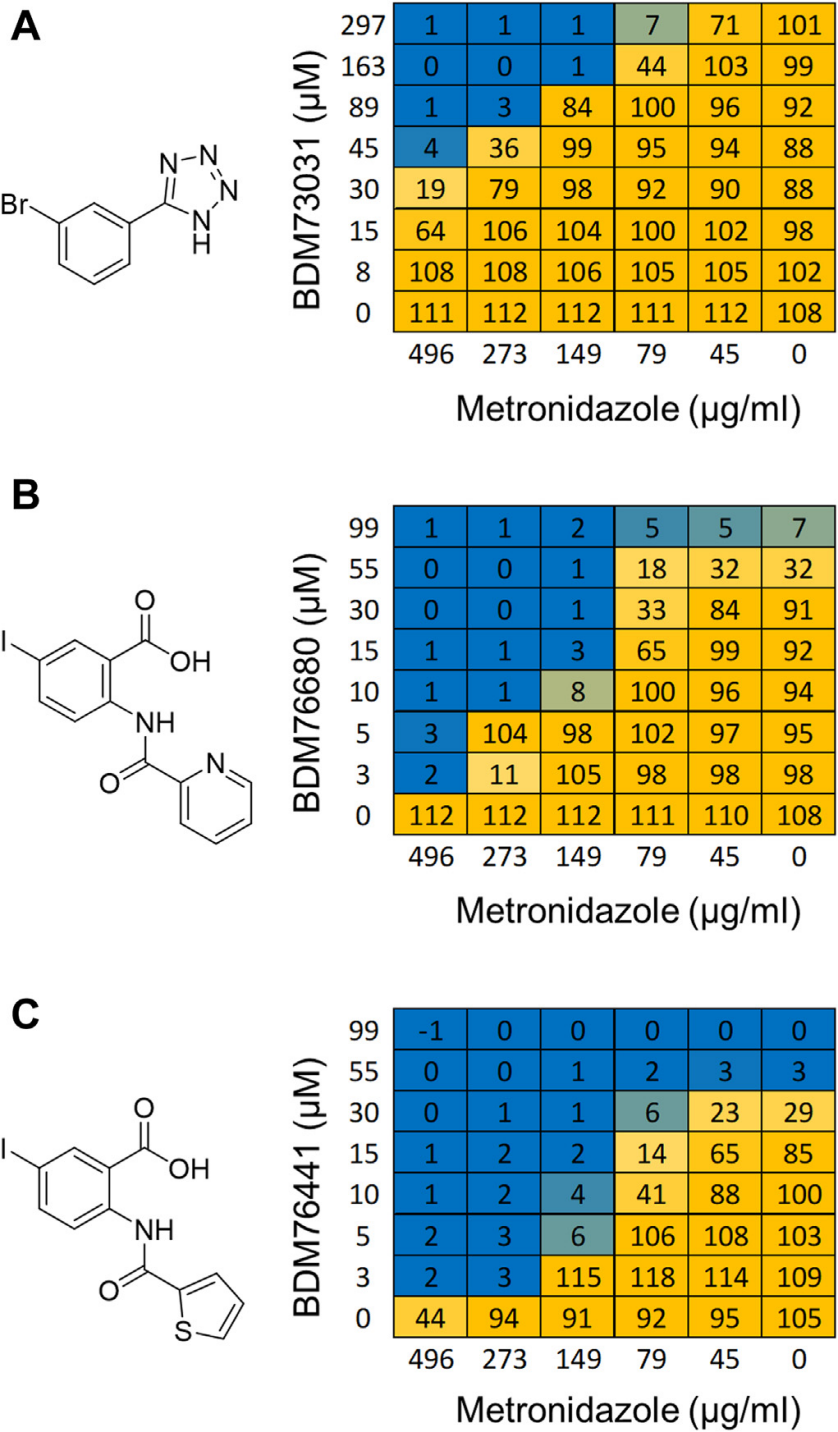
**Chemical structure of the identified MTZ boosters.***A*–*C*, BDM73031 (*A*), BDM76680 (*B*), and BDM76441 (*C*), as well as checkerboard assays demonstrating their ability to synergize with MTZ. Checkerboard assays were performed in microtiter plates with *E. coli* Δ*tolC* grown in liquid culture under standard aerobic conditions. Numbers represent percentage resazurin turnover after 5 h of compound exposure (as a surrogate of bacterial viability). Checkerboard assays for BDM76441 were also repeated on solid media as shown in Fig. S1. MTZ, metronidazole.

MTZ was confirmed to be inactive against both wildtype (WT) and efflux-deficient (Δ*tolC) E. coli* strains under aerobic culture conditions (using liquid culture in microplate assays) up to concentrations of 250 to 500 μg/ml (>1.5 mM). To identify molecules that potentiate MTZ antibiotic activity in such aerobic conditions, a chemical library of 2117 in-house compounds consisting of fragments (screened at 300 μM) and small molecules designed to target mycobacterial transcription factors (screened at 30 μM) were tested alone and in combination with an inactive dose of MTZ (50 μg/ml, 292 μM) against *E. coli* BW25113 and its Δ*tolC* derivative. No molecule was found to synergize with MTZ on the *E. coli* WT strain. In contrast, on the efflux-deficient strain, two molecules showed antibiotic activity in combination with MTZ, but not alone. These hits contained a small brominated phenyl tetrazole fragment, **BDM73031,** (structure in Fig. 1, no intrinsic activity at 300 μM but prevented growth in the presence of 50 μg/ml MTZ) and a halogen substituted anthranillic acid molecule, **BDM76680** (structure in Fig. 1, no intrinsic activity at 30 μM but significantly impacted bacterial viability in the presence of 50 μg/ml MTZ). Interestingly, both **BDM73031** and **BDM76680** share a similar pharmacophore as the tetrazole group of **BDM73031** is an isostere of carboxylic acid. Following resynthesis of **BDM73031** and **BDM76680,** both molecules were confirmed to potentiate MTZ (50 μg/ml) activity, though lethal synergy required 300 μM of **BDM73031** or 30 μM of **BDM76680**.

To identify more potent adjuvants of MTZ activity, 42 in-house halogen substituted anthranillic acid analogs were evaluated, among which six were found to boost MTZ activity at a lower concentration than 30 μM, though some also showed significant intrinsic antibiotic activity. The most potent anthranillic acid analog was **BDM76441,** for which 5 μM was sufficient to potentate the activity of 50 μg/ml of MTZ to prevent bacterial growth. Checkerboard assays were then performed on *E. coli* Δ*tolC* to substantiate the observed synergy between MTZ and the three MTZ potentiators **BDM73031, BDM76680,** and **BDM76441** (Fig. 1). These data confirmed that in the absence of the booster, MTZ had no antibiotic activity [minimum inhibitory concentration (MIC)_90_ >500 μg/ml], while in combination with **BDM73031, BDM76680,** or **BDM76441,** antibiotic activity decreased to 79 μg/ml (Fig. 1). In these assays, no intrinsic antibiotic activity was observed for **BDM73031,** while **BDM76680** (at 99 μM) and **BDM76441** (at 55 μM) had some antibiotic activity, though only at concentrations 10- to 20-fold greater than those needed to potentiate MTZ, suggesting a synergistic rather than an additive effect (Fig. 1). To verify that this synergy occurred under true aerobic conditions, rather than putative microaerophilic conditions in the liquid culture, checkerboard assays were also performed with *E. coli* Δ*tolC* grown on solid media spiked with MTZ/**BDM76441** combinations, with data confirming synergy (Fig. S1). MTZ/**BDM76441** checkerboard assays showed no MTZ boosting in the parental efflux competent *E.coli* strain BW25113, neither on other *E. coli* strains (Fig. S2), suggesting that further optimization of this anthranillic acid–based chemical series would be needed to overcome the penetration barrier of *E. coli*. **BDM76441** itself showed no apparent cytotoxicity on BALB/3T3 cells at a concentration tested of 100 μM over 48 h as measured by Hoescht and propidium iodide viability staining.

### BDM76441 can potentiate other nitro-aromatic prodrugs

To evaluate whether the identified MTZ boosters could potentiate the activity of other nitroaromatic antibiotic prodrugs, the impact of **BDM76441** was tested on the susceptibility of *E. coli* Δ*tolC* to a panel of commercially available molecules. We found that many of these nitroaromatic prodrugs were neither active alone nor in the presence of **BDM76441** (Table S1). Nonetheless, among the compounds that showed some antibiotic activity, co-exposure with **BDM76441** potentiated the antibiotic activity of 2-methyl-4-nitroimidazoles (close MTZ analogs) but not that of the nonmethylated 4-nitroimidazole (benznidazole) or the nitrofuran analogue (nitrofurazone) (Table S1). These results suggest that the boosting capacity of **BDM76441** is not universal toward nitroaromatics but instead is limited to MTZ-related analogs with the 2-methyl-4-nitroimidazole pharmacophore.

### The three MTZ boosters promote a similar transcriptional dysregulation in E. coli ΔtolC

To gain insight into the potential mechanism by which the MTZ boosters may act, the transcriptomic response of *E. coli* Δ*tolC* exposed to MTZ boosters was analyzed by RNAseq. The booster concentrations chosen for this study caused synergy with MTZ and were well below their intrinsic antibiotic activity [**BDM73031** (150 μM), **BDM76680** (15 μM {one-sixth MIC}), and **BDM76441** (6 μM {one-sixth MIC})]. Transcriptome data showed that all three compounds elicited a similar response in *E. coli* Δ*tolC*, with 61 common upregulated and 22 common downregulated genes (Fig. S3 and Table S2). A major common transcriptional response identified was the overexpression of the multiple antibiotic-resistance *marRAB* operon (*mar* operon), accompanied by the dysregulation of 20 MarA-regulated genes (17), including *acrA/B*, *inaA*, *yadG/H/I, nfsA,* and *nfsB* (Table S2). In addition, a plethora of genes implicated in glutathione biosynthesis (18) were overexpressed, including pathways for thiosulfate (*cy*s*A*/*W/T/P, yeeD/E)*, sulfate (*sbp, cysC/N/D/H/I/J*), and L-cystine (*yecC/S, dcyD, and ydjN*) assimilation, the glutathione synthetase (*gshA*), and uptake systems for glutathione (*gsiA/B*).

### MTZ boosting requires the upregulation of the *mar* operon

**Figure 2:**
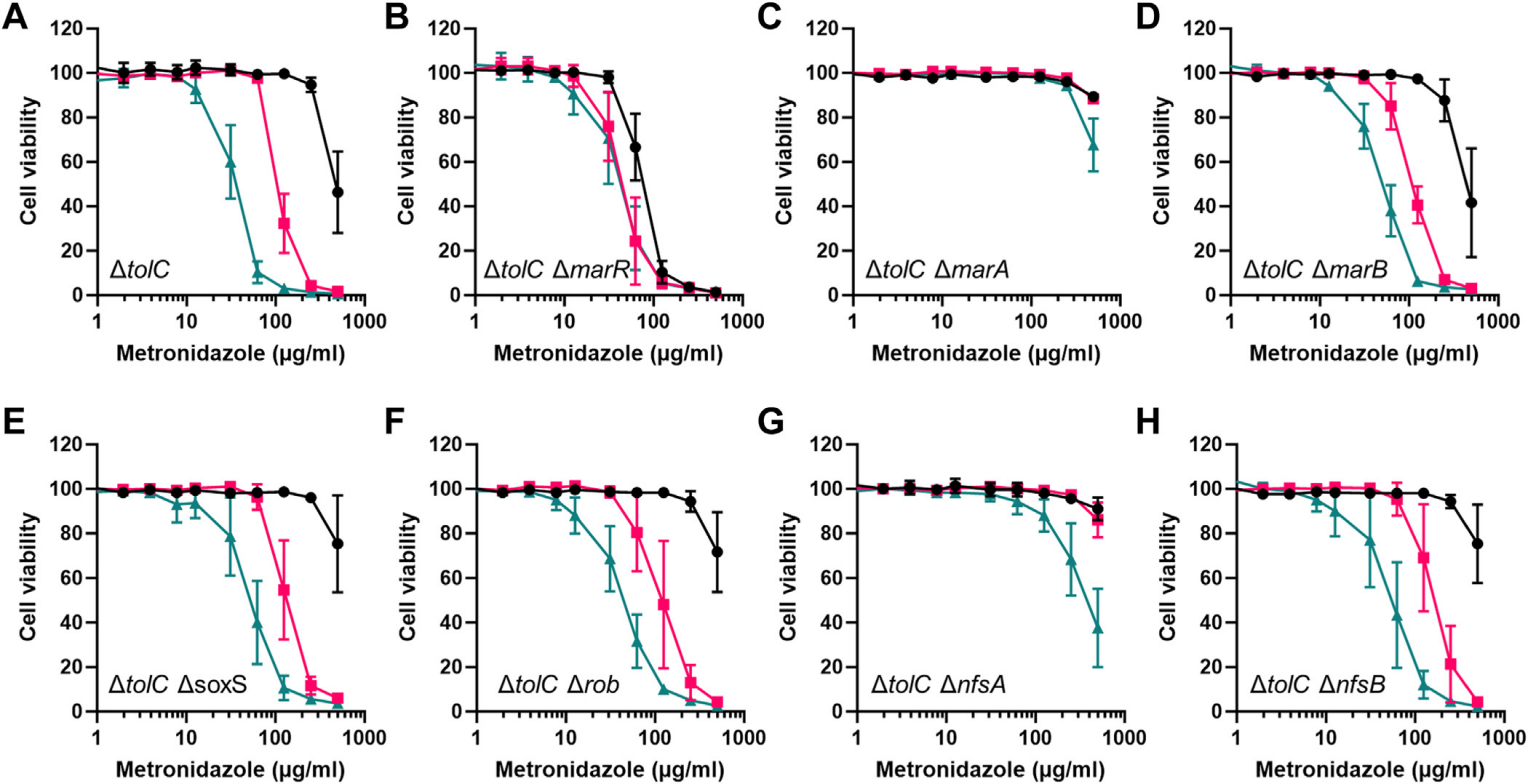
**BDM76441 effect on MTZ antibiotic activity in *E. coli strains.****A*–*H*, Δ*tolC* (*A*), Δ*tolC* Δ*marR* (*B*), Δ*tolC* Δ*marA* (*C*), Δ*tolC* Δ*marB* (*D*), Δ*tolC* Δ*soxS* (*E*), Δ*tolC* Δ*rob* (*F*), Δ*tolC* Δ*nfsA* (*G*), and Δ*tolC* Δ*nfsB* (*H*). Bacterial viability values are shown for MTZ alone (*black lines*) or in combination with 2 μM (*red*) or 6 μM of **BDM76441** (*green*). The values and error bars represent the means and ranges from three independent experiments. MTZ, metronidazole.

The transcriptome analysis on *E. coli* Δ*tolC* found the MTZ boosters to induce the *marRAB* operon, but as this operon is upregulated by many xenobiotics, it was unclear if this upregulation was directly associated with MTZ boosting. Transcription of the *marRAB* operon is regulated by MarR (autorepressor), by MarA (autoactivator) as well as by other genome-wide regulators such as SoxS, Rob, Fis, AcrR, and Cra (19, 20, 21). To investigate if upregulation of the *mar* operon was required in MTZ boosting and to identify the principle transcriptional regulators involved, we constructed derivatives of the *E. coli* Δ*tolC* strain deleted for the *marR, marA, marB, soxS,* or *rob* genes. Firstly, MTZ susceptibility assays of these mutant strains (in the absence of the MTZ boosters) showed that the deletion of *marR* (with *mar* operon overexpression as a result) led to MTZ sensitization and that the deletion of *marA* slightly increased resistance to MTZ. In contrast, the deletion of *marB, soxS,* or *rob* had no impact (Fig. 2). Next, we observed that the deletion of *marR* or *marA* also greatly reduced **BDM76441-**mediated boosting of MTZ activity, while this was not the case when *marB, soxS,* or *rob* were deleted (Fig. 2). Taken together, these data indicate that both MarR and MarA play a major role in mediating **BDM76441** boosting of MTZ in *E. coli* Δ*tolC*. As MarR represses *marA* expression, it would seem likely that **BDM76441** could interact with MarR and affect its activity.

### BDM76441 binds MarR and prevents its binding to the marRAB promoter

**Figure 3:**
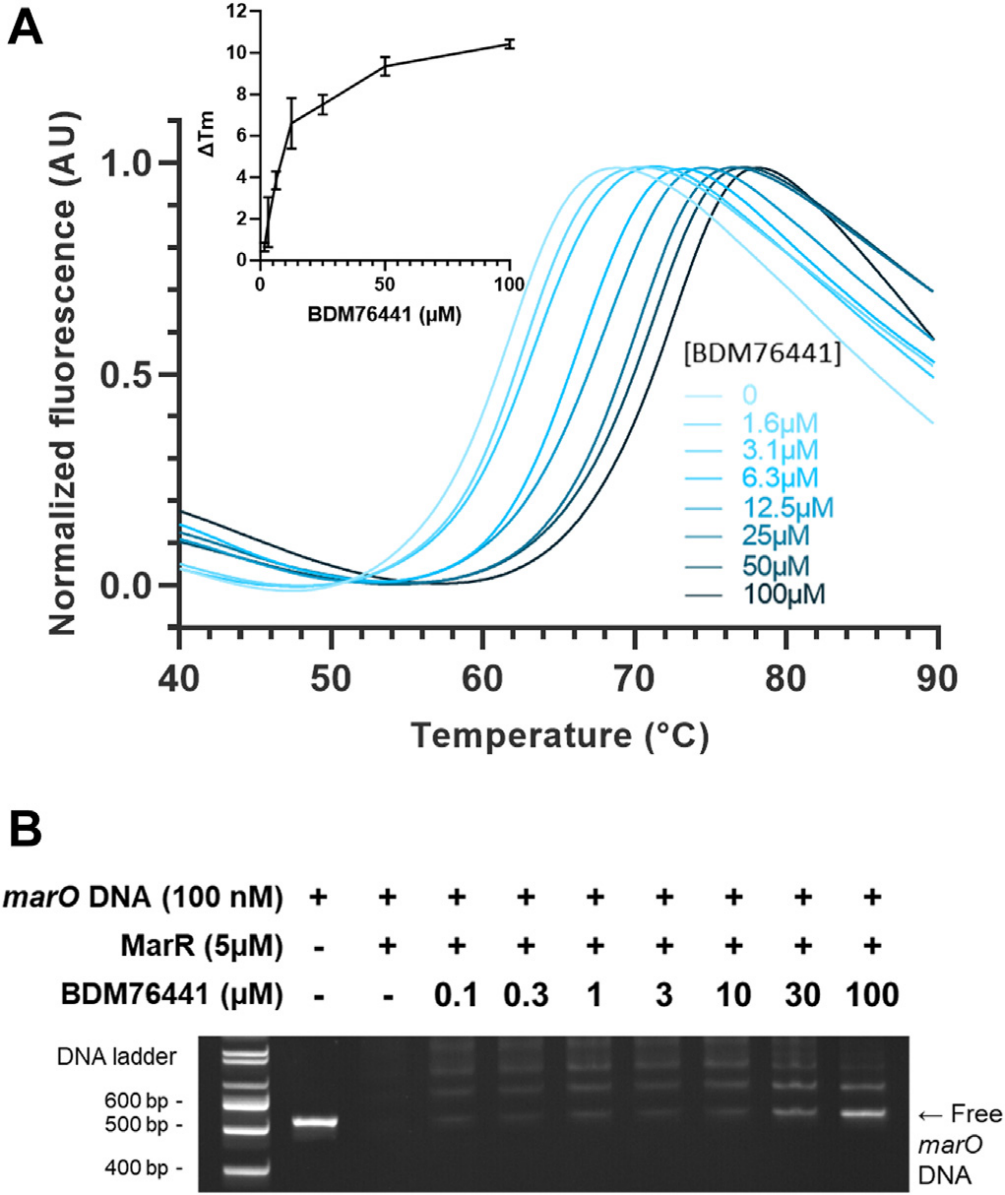
**BDM76441 binds MarR and prevents MarR binding to DNA.***A*, thermal shift assay of purified recombinant MarR in the presence of increasing concentrations of **BDM76441** shows a thermal stabilization of the transcriptional repressor. The inlay presents the **BDM76441** concentration-dependent change in thermal stability. *B*, a representative EMSA agarose gel with ethidium bromide staining for DNA (source image shown in Fig. S4C). In the absence of MarR, the PCR product bearing the *marRAB* promoter (*marO*) is clearly visible, while it is retarded in the presence of MarR. The addition of **BDM76441** leads to a dose-dependent release of *marO*. Source images of this and replicate EMSA gels are shown in Fig. S4. EMSA, electrophoretic mobility shift assay; *marO*, *marRAB* promoter.

To test if **BDM76441** could bind the MarR repressor, thermal shift assays were conducted with purified recombinant MarR and **BDM76441** (0–100 μM final). We observed that **BDM76441** mediated a concentration-dependent increase in the thermal stability of MarR (ΔTm = 10.4 °C), with an apparent binding affinity (Kd) of 9.8 ± 1.6 μM (Fig. 3*A*). We then used electrophoretic mobility shift assays to evaluate the impact of **BDM76441** on the binding of MarR to the *marRAB* promoter (*marO*). These data showed that **BDM76441** caused a concentration-dependent release of the *marO*-bearing DNA fragment from MarR with an average EC_50_ of 38 μM (Figs. 3*B* and S4). Together, these *in vitro* results suggest that **BDM76441** could bind MarR *in vivo* and thus prevent MarR repression of the *marRAB* promoter, leading to the upregulation of the *mar* operon and MarA production.

### Cys80 of MarR is not required for BDM76441-mediated MTZ boosting

MarR is modulated by salicylic acid, a molecule that shares a pharmacophore part similar to that of the anthranillic acid MTZ modulators. Interestingly and in support of the work presented here, salicylic acid at high concentrations (700 μM) was also found to boost MTZ activity in *E. coli* Δ*tolC* (data not shown). While salicylic acid was originally described to directly interact with MarR homodimers (22, 23), later reports suggested that MarR inactivation by salicylic acid was indirect and occurred through copper(II)-induced interprotomer disulfide bonds formation between the MarR cysteine 80 residues (Cys80) (24). To evaluate the role of MarR Cys80 in the boosting activity of **BDM76441**, the *marR* Cys80 codon was mutagenized to encode Ser in the chromosomal gene of *E. coli* Δ*tolC*. The MTZ boosting in the resulting *E. coli* Δ*tolC marR(C80S)* strain by **BDM76441** remained constant (Fig. S5), showing that Cys80 is not required for **BDM76441** boosting.

### Transcriptomic signature of E. coli ΔtolC exposed to MTZ and MTZ potentiator

To understand how *marRAB* induction mediated by MTZ boosters leads to the potentiation of MTZ antibacterial activity, transcriptome analysis was performed on aerobic cultures of *E. coli* Δ*tolC* exposed to a low concentration of MTZ (0.1 mg/ml) in combination with a sub-MIC concentration of **BDM76441** (6 μM) or **BDM73031** (150 μM) and compared to gene expression in cultures grown with MTZ or boosters alone (described above). RNAseq-based differential analysis identified 41 genes for which transcription was specifically dysregulated (4 downregulated, 37 upregulated) in combinations of MTZ with **BDM76441** or **BDM73031** (Table S3). Strikingly, this dysregulation was almost entirely linked to the DNA damage SOS response of the LexA regulon (29 affected genes). As an additional control, we also studied the transcriptome of *E. coli* Δ*tolC* exposed to a high concentration of MTZ (2 mg/ml) affecting bacterial growth, and we observed a very similar LexA-mediated DNA damage stress response signature (Fig. S6). Together these results suggest that under aerobic conditions, our MTZ boosters induce MTZ to cause DNA damage. DNA damage signatures have also been reported when MTZ is activated by either the PFOR, the FrxA or RdxA nitroreductases in *H. pylori* (25, 26), or the thioredoxin reductase in *T. vaginalis* and *G. lamblia* (27, 28) under anaerobic or microaerophilic conditions.

### Induction of the NfsA nitroreductase is required for BDM76441 boosting of MTZ activity

**Figure 4:**
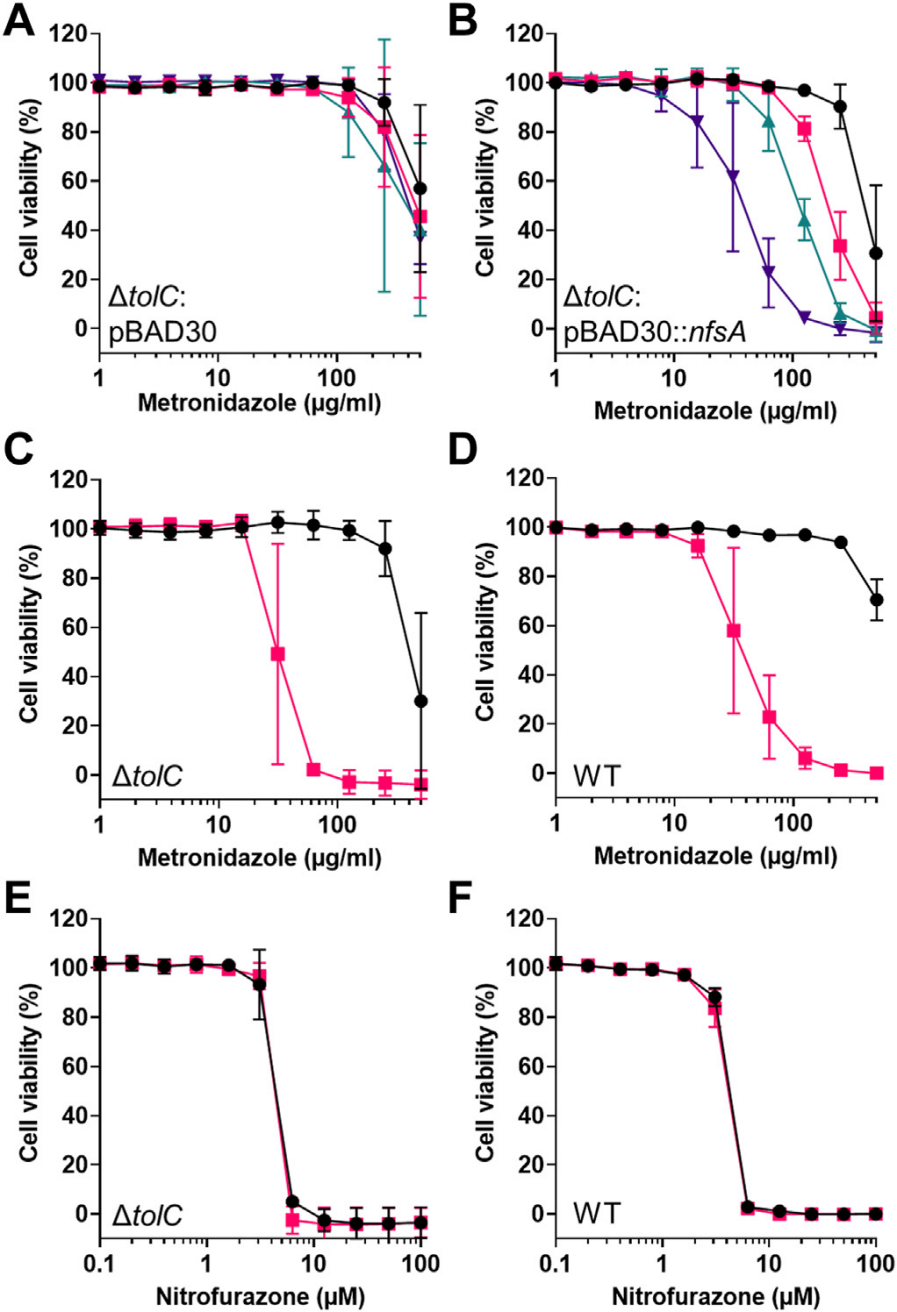
**Metronidazole and nitrofurazone activity on *E. coli* strains overproducing or not NfsA.***A* and *B*, metronidazole activity on *E. coli* Δ*tolC* containing pBAD30 (*A*) or pBAD30::*nfsA* (*B*), in the absence of arabinose (*black*) or with 0.1% (*pink*), 0.5% (*green*), or 5% arabinose (*purple*). *C* and *D*, metronidazole activity on *E. coli* Δ*tolC* (*C*) or WT *E. coli* (*D*), bearing empty pUC18 (*black*) or pUC18::*nfsA* (*pink*). *E* and *F*, nitrofurazone activity on *E. coli* Δ*tolC* (*E*) or WT *E. coli* (*F*), bearing empty pUC18 (*black*) or pUC18::*nfsA* (*pink*). Values and ranges are from three independent experiments.

Our transcriptomic data showed that the NfsA and NfsB nitroreductases were both upregulated following exposure to the MTZ boosters (Table S1). Their encoding genes, *nfsA* and *nfsB*, both belong to the MarA regulon (17). In addition, NfsA has been shown to induce MTZ-mediated DNA damage when expressed in eukaryotic cells (29). Together, these data suggested that NfsA and/or NfsB could play a role in boosting MTZ antibiotic activity. To test this hypothesis, the impact of *nfsA* or *nfsB* deletion was examined. Deletion of *nfsA* or *nfsB* in *E. coli* Δ*tolC* was found to have no major impact on the basal susceptibility to MTZ (Fig. 2, *G* and *H*); however, the absence of *nfsA* (but not *nfsB*) prevented the potentiation of MTZ susceptibility by **BDM76441** (Fig. 2, *G* and *H*). To confirm that NfsA overexpression facilitated MTZ activation, *nfsA* was overexpressed in *E. coli* Δ*tolC* using either an inducible pBAD30 expression system or a constitutive pUC18 expression system. In *E. coli* Δ*tolC* (pBAD30::nfsA), arabinose-mediated overexpression of *nfsA* led to marked sensitization to MTZ (Fig. 4), while *nfsB* overexpression in *E. coli* Δ*tolC* (pBAD30::nfsB) mediated only a slight potentiation of MTZ activity (Fig. S7). Regarding *E. coli* Δ*tolC* (pUC18::nfsA), where *nfsA* is expressed under a strong promoter, the strain was even more sensitive to MTZ (MIC_90_ of 62.5 μg/ml) compared to the empty vector control strain (MIC_90_ > 500 μg/ml) (Fig. 4). Interestingly, WT *E. coli* transformed with pUC18::nfsA also became sensitive to MTZ activity (Fig. 4*D*), suggesting that high NfsA activity drives MTZ activation and that efflux limits the entry of the MTZ boosters. These genetic experiments clearly show that *nfsA* overexpression can potentiate the antibiotic activity of MTZ in aerobic conditions and that NfsA is the primary nitroreductase involved in **BDM76441**-mediated MTZ potentiation.

The nitrofuran antibiotic nitrofurazone (NFZ) was originally validated as a substrate for reduction by NfsA (30). For this reason, the impact of *nfsA* overexpression was also tested on NFZ susceptibility; however, we found that *E. coli* Δ*tolC* (pUC18::nfsA) and *E. coli* Δ*tolC* (pUC18) had similar NFZ susceptibility (Fig. 4, *E* and *F*). This indicated that while NfsA can reduce NFZ, it is not the primary activator of NFZ antibiotic activity in *E. coli*.

### Evaluation of the biochemical reduction of MTZ and NFZ by recombinant NfsA

NfsA is described as an oxygen-insensitive NADPH-dependent flavin oxidoreductase capable of metabolizing a plethora of nitroaromatic compounds (30). While NfsA is considered as a 2-electron transfer nitroreductase, a previous study reported that NfsA is capable of reducing ferrocyanide through 1-electron transfer (30). Our initial experiments with purified recombinant NfsA corroborated these enzyme characteristics and confirmed that NfsA could reduce both menadione (2-electron reduction) and ferrocyanide (1-electron reduction) (Fig. S8).

**Figure 5:**
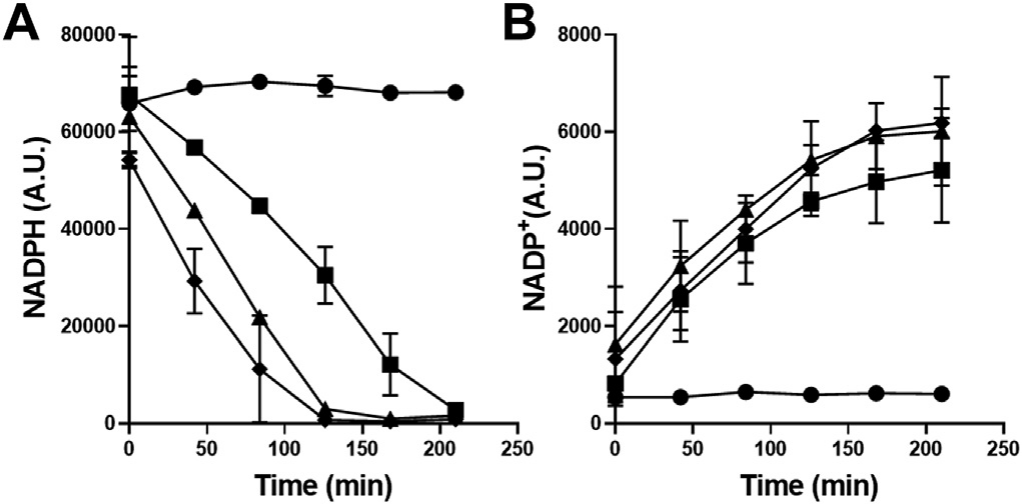
**Kinetics of NfsA activity on MTZ and NFZ.***A* and *B*, NADPH or NADP+ concentrations followed over time in reaction mix containing NADPH only (*circle*); NfsA and NADPH (*square*); NfsA, MTZ, and NADPH (*triangle*); or NfsA, NFZ, and NADPH (*diamond*). Cofactor concentration was evaluated overtime by negative mode mass spectrometry analysis at m/z 744 for NADPH or 741 for NADP^+^. Values and ranges are from three independent experiments. MTZ, metronidazole; NFZ, nitrofurazone.

In biochemical assays with purified recombinant NfsA, we observed a rapid biotransformation of NFZ accompanied by a conversion of NADPH to NADP+ as measured by UHPLC-MS analysis (Fig. 5 and Fig. S9). With this setup, no clear NFZ metabolites could be detected by UHPLC-MS; however, when performed in the presence of reduced glutathione (GSH), to capture potential reactive intermediates, two products were observed with a mass of m/z^+1^ of 474 and 490 Da (Figs. S10 and S11). LC-MS/MS fragmentation analysis of these products corresponded to the glutathione conjugate of a NFZ-nitroso metabolite (-hydroxysulfenamide) (m/z^+1^ = 490) and the sulfinamide (m/z^+1^ = 474) (31) (Figs. S12 and S13), pointing to NfsA-mediated 2-electron reduction of NFZ.

In contrast, when the NfsA biochemical assays were performed with MTZ and NADPH, no significant decrease in MTZ concentration was observed by UHPLC-MS (Fig. S14), and when experiments were repeated in the presence of GSH, no glutathione conjugate could be detected (Figs. S15 and S16). Despite this apparent lack of activity toward MTZ, a moderate decrease in NADPH and an increase in NADP+ was observed, that was greater than that observed without MTZ (Fig. 5). This indicated that while no 2-electron reduction metabolites were being formed, NADPH was slowly being consumed in the reaction with potential (alternative) undetected electron transfer to MTZ. We thus hypothesized that NfsA could mediate 1-electron reduction of MTZ to the MTZ radical anion (MTZ·^-^), which could then revert back to the parental MTZ by losing the electron to the reaction mixture, thus not impacting the overall amount of MTZ in the reaction mixture.

### Electrochemical reduction potential of MTZ and NFZ

**Figure 6:**
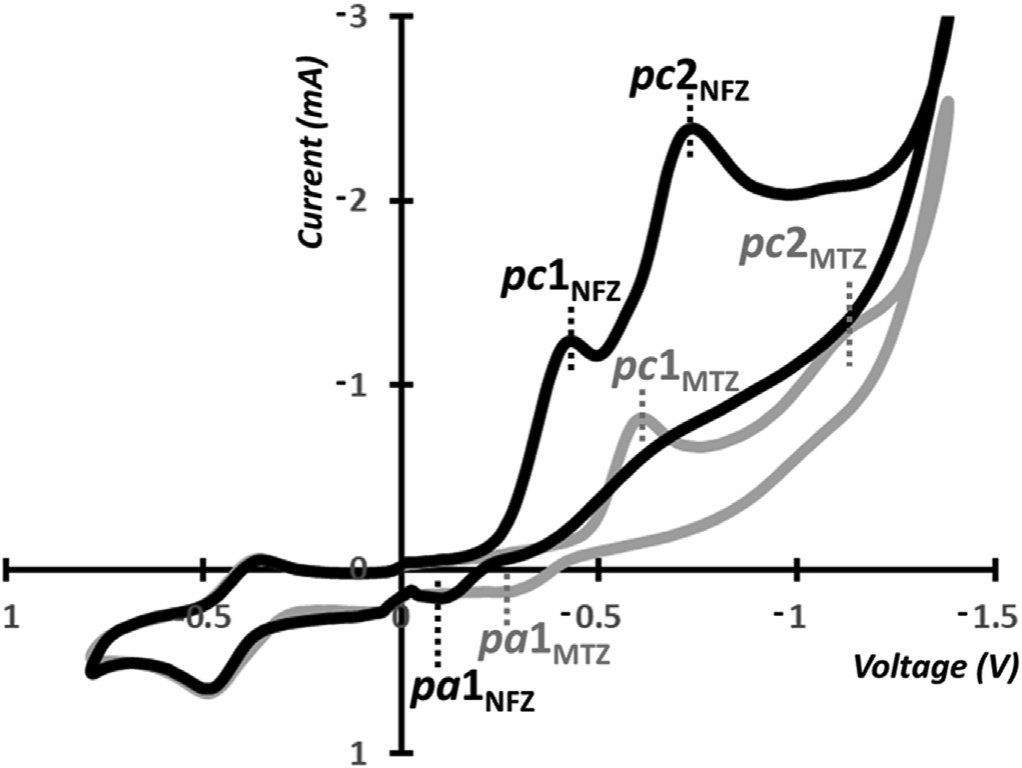
**Cyclic voltammmograms of MTZ and NTZ.** MTZ (2 mM, *grey*) and NFZ (2 mM, *black*) standardized against the 1-electron Fc/Fc+ redox couple in 0.5 M citrate buffer (pH 7.4)/dimethylformamide (3/2, v/v) against Ag/Ag+ system, T = 25 °C, sweep: 100 mV/s, 0 V starting point. MTZ, metronidazole; NFZ, nitrofurazone.

Previous studies investigating the reduction potential of MTZ and NFZ by cyclic voltammetry (CV) showed that MTZ requires more energy for reduction than NFZ, although these experiments were not conducted under identical conditions, especially with respect to solvents (32, 33, 34, 35). To allow for a direct comparison, we decided to perform CV measurements using a common citrate buffer (pH 7.4) and dimethylformamide mixture (3/2 v/v) in which both MTZ and NFZ were soluble. The CV experiments were carried out with and without ferrocene (36) as an internal 1-electron reference system. In the case of NFZ, CV measurements identified two distinctive reduction peaks (Fig. 6) similar to that reported previously (33), with Pc1_NFZ_ (at −0.43 V) representing 1-electron reduction to the NFZ radical anion and pc2_NFZ_ (at −0.75 V) representing an additional 3-electron reduction to the NFZ hydroxylamine. In a similar fashion, two distinctive reduction peaks were observed for MTZ (Fig. 6) as was reported previously (32), with a 1-electron reduction peak pc1_MTZ_ (at −0.78 V) corresponding to the formation of the MTZ radical anion, and the second reduction peak, pc2_MTZ_ (around 1.2 V) likely showing the formation of the hydroxylamine MTZ reduction product (32). The classical F_c_/F_c+_= redox couple was used as an internal reference in both cases. From the obtained results, we can conclude that MTZ is less electroactive than NFZ, requiring greater reducing conditions.

### Validation of MTZ radical anions formation by NfsA

**Figure 7:**
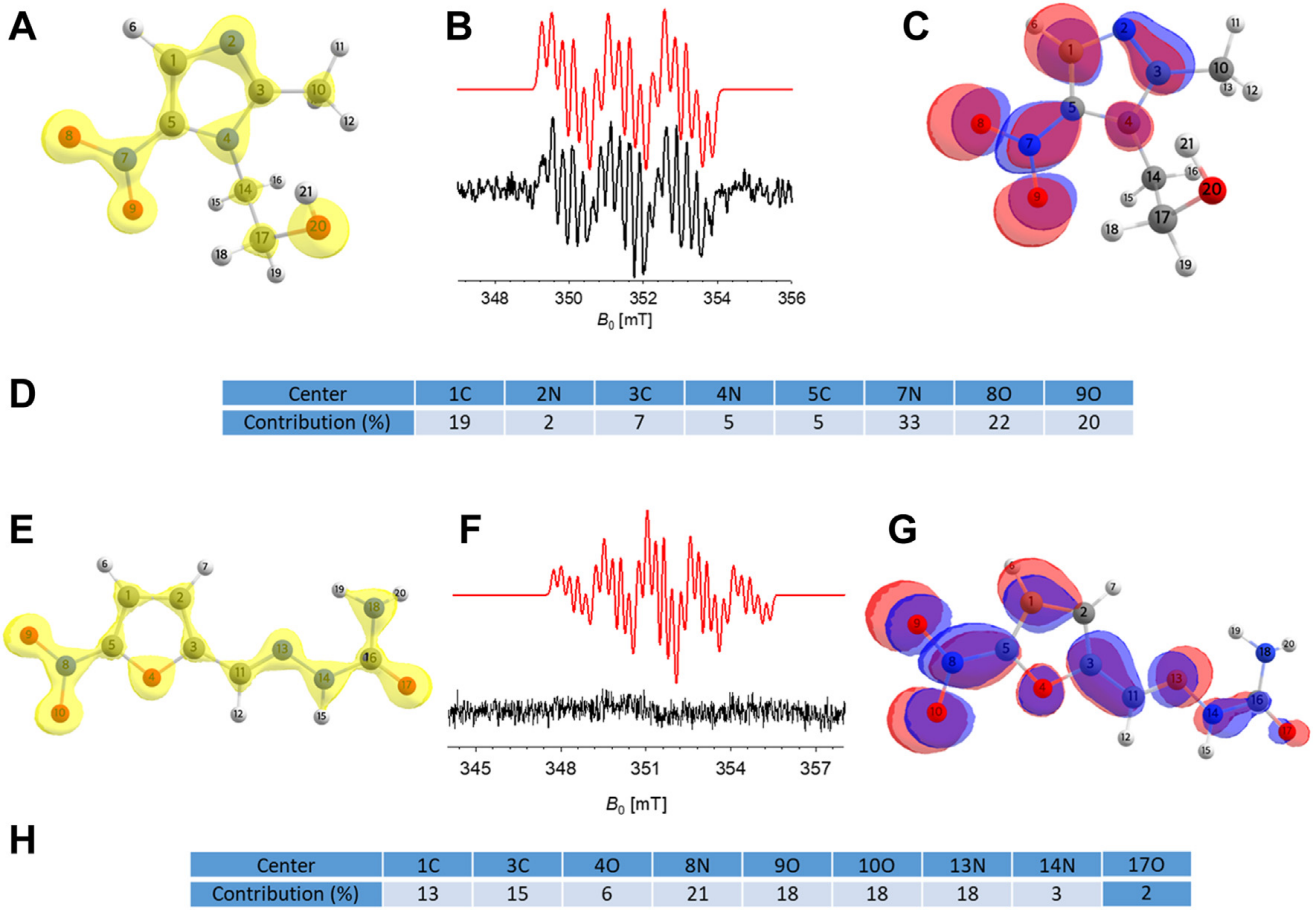
**NfsA mediated formation of radical anion intermediates from MTZ *versus* NFZ.***A* and *E*, images of the spin densities for MTZ (*A*) or NFZ (*E*) as obtained by DFT calculation according to the hyperfine coupling constants reported on supporting information (Tables S4 and S5 respectively). *B* and *F*, EPR spectra showing the observed CW EPR spectrum (*black line*) for MTZ (*B*) or NFZ (*F*), with a corresponding simulated EPR spectrum for the radical anion species (*red line*). EPR spectra were recorded under aerobic conditions with the following condition: NfsA (1 μM), MTZ, or NFZ (100 μM) and NADPH (200 μM), averaging 64 scans for each experiment, with additional details in the Experimental procedures section. Hyperfine couplings of the MTZ fitted spectra [*red line*, a_iso_(^14^N, NO_2_) = 42.7 MHz, a_iso_(^1^H, H-4) = 17 MHz, a_iso_(^1^H, CH_3_) = 7.33 MHz], as well as g-value [g_iso_ = 2.0043(4)] are in agreement with data reported in literature (10). Data clearly show concordance between the observed and simulated spectra for MTZ radical anion formation, while this is not the case for NFZ. *C*, *G*, *D* and *H*, images showing the spin density plots (*left* contour plots) and the SOMOs (*right* contour plots) for the MTZ (*C*) or NFZ (*G*) radical anion, including a table for the individual contribution of each center [%] to the SOMO for each species (*D* and *H*). These images show the spatially localized nature of the MTZ radical in contrast to the electronic delocalization observed for the NFZ radicals having the spin density equally distributed on all atoms. DFT, density functional theory; EPR, electron paramagnetic resonance; MTZ, metronidazole; NFZ, nitrofurazone; SOMO, singly occupied molecular orbital.

Electron paramagnetic resonance (EPR) has previously been used to demonstrate the formation of MTZ radical species following the incubation of MTZ with *T. vaginalis* extracts under strictly anaerobic conditions (10, 11, 12). We thus used EPR to evaluate whether NfsA was able to mediate the formation of MTZ radical anions under aerobic conditions. Indeed, when recombinant NfsA was incubated with MTZ and NADPH under aerobic conditions, the detected EPR signal was identical to that shown by Hrdý *et al.* (10) for the formation of MTZ radical anion under anaerobic conditions (Fig. 7). Specifically, the EPR spectrum showed strong coupling of the ^14^N (of the MTZ NO_2_ group) with two coupled ^1^H nuclei (Fig. 7*B*, black line), in line with calculated hyperfine coupling constants obtained by density functional theory (DFT) calculated hyperfine values for the MTZ radical anion (Fig. 7*D* and **7***B*, red line). To further validate that the observed EPR signal originated from the MTZ radical anion, EPR experiments were repeated using a C^13^-labeled isotopolog of MTZ (Fig. S17). While this EPR spectrum exhibited a lower signal-to-noise ratio, the main spectral features were maintained and in agreement with the calculated hyperfine coupling constants obtained by DFT calculations when the corresponding MTZ nuclei were replaced with ^15^N and ^13^C isotopes. Overall, these results confirmed the formation of MTZ radical anions by NfsA under aerobic conditions.

In contrast, in EPR experiments performed on NFZ in the presence of NfsA and NADPH, the reaction mixture underwent a rapid color change indicating a rapid metabolism of the compound. In addition, we detected no signal corresponding to NFZ radical anion such as that observed using crude *E. coli* nitroreductases extracts under anaerobic conditions (37). Taken together, these data suggest that in the timeframe of the EPR experimental setup, NFZ radical anions are not readily detected, and instead NfsA catalyzes two or more electron reduction of NFZ, in agreement with the biochemical results described above.

### Calculated spin densities and hyperfine coupling constants of MTZ and NFZ radical anions

DFT calculations were performed to predict the electronic structures of the MTZ and NFZ radical anions based on Mulliken spin populations and generated singly occupied molecular orbital (SOMO) plots shown in Figure 7. These plots confirmed that for the MTZ radical anion, the spin density distribution was confined between the nitro-functional group and the C1-H6 as shown in Fig. 7*C*, with calculated contributions (%) of the SOMO shown in Fig. 7*D*. For the NFZ radical anion (Fig. 7, *E* and *H*), the predicted spin density distribution and SOMO were more delocalized than for the MTZ radical anion. Together, this helps to explain the difference in the reduction potentials of MTZ and NFZ, where the more confined electron distribution on the MTZ radical anion would require more energy for the second electron transfer.

## Discussion

**Figure 8:**
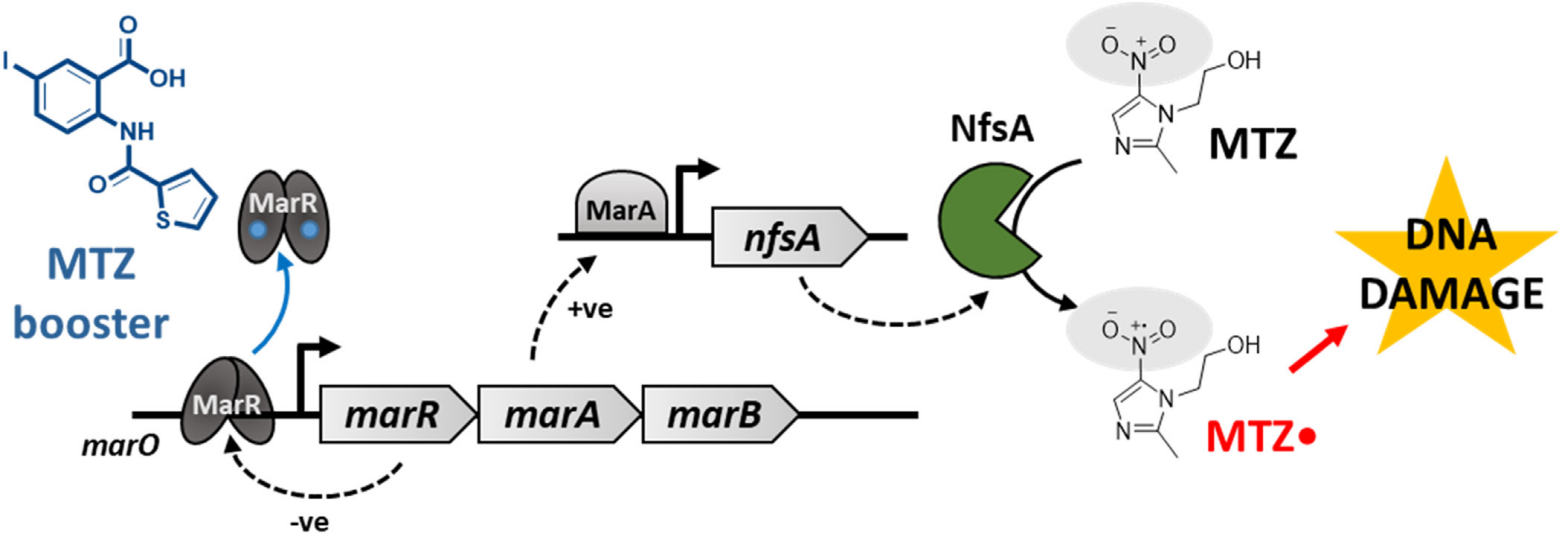
**Summary schematic of the uncovered mechanism by which anthranillic acid analogs mediate the boosting of MTZ prodrug activity in *E. coli* Δ*tolC* under aerobic conditions.***marO*, *marRAB* promoter; MTZ, metronidazole.

The research presented here provides proof-of-concept evidence that small molecules, such as anthranillic acid analogs, can boost the prodrug activity of MTZ in *E. coli* under aerobic conditions. We showed that this is achieved by the anthranillic acid analog binding the MarR repressor, preventing its binding to the *mar* promoter and thus leading to overexpression of the *marRAB* operon. The resulting upregulation of the MarA activator induces the overexpression of the gene encoding the nitroreductase NfsA. This flavin-containing enzyme then mediates a 1-electron reduction of MTZ to the toxic MTZ radical anion that causes DNA damage (Fig. 8). Together, this highlights the prospect of using small molecules to improve the antibiotic activity of prodrugs in *E. coli* and highlights the fact that MTZ antibiotic activity can be achieved under aerobic conditions.

Since its development in the 1950s, the antibiotic prodrug MTZ has been widely used with a good safety profile for the treatment of some microaerophilic and anaerobic protozoan and bacterial infections. Despite this long history, the main active intermediates of MTZ bioactivation in these microorganisms are not clearly identified. It is well established that MTZ reduction can proceed either *via* a 1-electron transfer to form reactive MTZ radical anions (as by PFOR) or *via* a 2-electron reduction to form reactive nitroso intermediates (such as by flavin reductases). In addition to this differential bioactivation, it has been argued that microaerophilic or anaerobic conditions are essential for MTZ activity, as aerobic conditions were argued to allow for the re-oxidation and neutralization of MTZ radical anions in a “futile cycle”. An important finding from our work is that in *E. coli,* MTZ bioactivation to MTZ radical anions can indeed be achieved under aerobic conditions. This suggests that under the boosted conditions, the “futile cycle” proposed to inactivate MTZ radical anions either is insufficient to completely inactivate all the MTZ• or that it generates an excess of superoxide anions which are themselves antibiotic.

An additional interesting finding from our work relates to rational prodrug antibiotic design. Previous data on PFOR-mediated MTZ activation and the data presented here point to radical intermediates as being important for the antibiotic activity of nitroaromatic prodrugs. This knowledge may facilitate the optimization of nitroaromatic prodrugs by fine-tuning their redox potential relative to that of the bioactivating reductase, for greater radical anions production.

Our data demonstrate that prodrug activation is achievable in gram-negative bacteria; however, there are certain obstacles to overcome to use this strategy as an adjuvant therapy. Firstly, the anthranillic acid analogs generated here only boost MTZ in efflux-deficient *E. coli*, probably because the adjuvants are effluxed in the WT background, however we observed that mechanistically, NfsA overexpression does improve MTZ activity in *E. coli* WT. An additional caveat is that induction of the *mar* operon is known to lead to increased expression of efflux pumps such as AcrAB-TolC, making the bacteria more resistant to many substrate antibiotics. On the other side, it is known that many xenobiotics elicit a stress response in *E. coli* including the induction of the *mar* operon and that mutations in MarR can be associated with antibiotic resistance. In such cases, MTZ or similar nitroaromatic prodrugs could be used to counter select such bacteria and synergize with current antibiotics, and such an approach would merit further investigation.

Together, this work has demonstrated that it is possible to use small molecules as transcriptional modulators to force the expression of prodrug activation enzymes in *E. coli* and thus boost MTZ antibiotic activity. In addition, this work has brought to light that MTZ activity is not restricted to low oxygen condition and that in aerobic conditions NfsA-mediated MTZ radical anions can kill *E.coli*.

## Experimental procedures

### Bacterial strains and plasmids

*E. coli* BW25113 and its Δ*tolC*732*::Km* derivative originated from the Keio Collection (38) and were obtained from the *E. coli* Genetic Stock Center. Bacteria were grown in cation-adjusted Mueller-Hinton broth (CAMHB; BD Difco) at 37 °C. *E. coli* Δ*tolC* double mutants CP100 (Δ*nfsA*), CP101 (Δ*nfsB*), CP102 (Δ*marR*), CP103 (Δ*marA*), CP104 (Δ*marB*), CP105 (Δ*soxS*), and CP106 (Δ*rob*) were constructed in EP664(pEP1436) by Red recombination (39) as described previously (40), with primers listed in Table S6.

*E. coli* Δ*tolC marR*-C80S was constructed by Red recombineering in two steps *via* an intermediate strain bearing the counter-selectable *marR*::Km1446 allele. First, RH1127 and RH1128 were used to PCR amplify the Km1446 kanamycin cassette from pEP1446 (40). Arabinose-induced EP664(pEP1436) electrocompetent cells were then transformed with the PCR product and selected on kanamycin plates at 28 °C. Correct integration of the Km1446 cassette into the *marR* gene was assessed by PCR. In the second step, arabinose-induced electrocompetent cells were prepared from BW25113 Δ*tolC marR:*:Km1446(pEP1436) and transformed with the *marR*-C80S PCR product generated with the RH1129-RH1130 primer pair on pET28-*marR*-C80S. Clones were selected at 28 °C on LB-Amp plates containing 200 ng/ml of anhydrotetracycline and then screened for Km sensitivity. Correct allelic exchange was confirmed by sequencing the RH1129-RH1130 PCR product. Plasmid pEP1436 was then cured on LB-5% sucrose plate at 37 °C.

To overexpress *nfsA* or *nfsB*, the genes were PCR amplified from BW25113 gDNA with primers RH980 and RH840 and RH968 and RH962, respectively (Table S6) and cloned into pBAD30 under the Para promoter using *XbaI* and *HindIII* restriction sites. The *nfsA* gene was also cloned into pUC18 under the Plac promoter.

To produce recombinant His-tagged MarR, the *marR* gene was amplified from BW25113 gDNA with primers RH842 and RH843 (Table S6) and cloned into pET15b using *NdeI* and *XhoI* restriction sites. The resulting plasmid was submitted to site-directed mutagenesis to yield pET15b-*marR*-C80s. To produce recombinant His-tagged NfsA, the *nfsA* gene was amplified from BW25113 gDNA with primers RH980 and RH840 (Table S6) and cloned into pET28 using *NdeI* and *SalI* restriction sites.

### Antibacterial activity

The MIC was determined using the colorimetric resazurin microtiter assay. Experiments were performed in 96-well plate format with a 100 μl final volume. Briefly, a suspension of an *E. coli* strain of interest was prepared in CAMHB (BD) at an *A*_600_ of 0.001. When required, boosters or arabinose were added to the suspension, and MTZ or NFZ antibiotics were tested in a concentration gradient through serial 2-fold dilutions. After 5 h of incubation at 37 °C, 10 μl of resazurin 12.5% was added to each well, and an additional incubation was carried out for 1.5 h. Fluorescence was measured using a Fluostar Optima BMG Labtech (Ex: 530 nm; Em: 590 nm). Values were converted to resazurin turnover based on minimal (no bacteria added to the medium) and maximal (no antibiotic added) fluorescence values. MIC_90_ values were determined when resaruzin turnover was ˂10% of control.

### Checkerboard assays

Checkerboard assays were performed in 384-well plate format. Boosters and MTZ (dissolved in DMSO) were distributed into the wells in a concentration gradient (from 496.5 μg/ml to 19.86 μg/ml for MTZ; from 296.7 μM to 5.935 μM for **BDM73031**; and from 99.3 μM to 1.99 μM for **BDM76680** and **BDM76441**) by an Echo liquid handler. The *E. coli* culture of interest was then diluted in CAMHB (BD) to an *A*_600_ of 0.001, and 45 μl of this suspension was added to each well using a VIAFILL liquid dispenser. After 5 h of incubation at 37 °C, 5 μl of resazurin was added to each well, and plates were further incubated of 1.5 h, 37 °C. Fluorescence was then measured as described above.

### RNA extraction and transcriptomic analysis

*E. coli* BW25113 ΔtolC::Km (38) was grown in CAMHB supplemented with 25 μg/ml of kanamycin for 3 h, and *A*_600_ was adjusted at 0.22. Aliquots of this culture (5 ml) were then incubated with each booster alone (**BDM73031**: 150 μM; **BDM76680**: 15 μM; **BDM76441**: 6 μM), with MTZ alone (100 μg/ml or 1 mg/ml), or with booster/MTZ combinations and incubated for 1 h at 37 °C with shaking. 1.25 ml of a 5/95 phenol/ethanol mixture was then added to the cultures to block RNA degradation. The bacteria were then pelleted and RNA extracted using 1 ml of TriReagent (Life Technologies) according to manufacturer’s instructions. RNA sequencing was performed by Genoscreen using a Hiseq 4000 run (Illumina) in paired-end reads (2 x 150 bp). Raw RNA-seq reads were processed with Illumina quality control tools using default settings. rRNA-specific reads were filtered out by mapping all the reads on *E. coli* BW25113 rRNA sequences using Bowtie2 (http://bowtie-bio.sourceforge.net/bowtie2/index.shtml). Analysis of the RNA sequencing data was conducted using Rockhopper v2.0.3 with the default parameters to calculate the reads per kilobase per million base pairs values for each coding sequence using the *E. coli* BW25113 (NZ_CP009273) genome annotation.

### Protein expression and purification

To produce recombinant His-tagged NfsA and MarR proteins, *E. coli* BL21 Rosetta (Novagen) was transformed with pET28a::*nfsA* or pET15b::*marR*. Strains were grown in LB at 37 °C to an *A*_600_ of 0.5, and protein production was induced for 3 h by addition of 1 mM IPTG. Cells were collected by centrifugation (6000 rpm, 30 min, 4 °C), and pellets were resuspended in buffer A (20 mM NaH_2_PO_4_, 500 mM NaCl, pH 7.4) containing 10 μg/ml of DNAse I and one tablet of complete Protease inhibitor cocktail (Roche). Cells were disrupted by sonication, centrifuged (12,000 rpm for 30 min at 4 °C), and the supernatant was used for protein purification. Purification was performed using an Äkta Purifier 900 FPLC system with a HisPur Cobalt Chromatography Cartridge 5 ml (Thermo Scientific). The cell-free extract was loaded onto the cartridge pre-equilibrated with buffer A, and proteins were eluted with a linear gradient to buffer A with 300 mM imidazole (over 30 min) and monitored by UV detection at 215 nm and 254 nm. One milliliter samples were collected and analyzed by SDS-PAGE analysis to identify fractions containing pure recombinant protein. Such fractions were concentrated using a Vivaspin turbo concentrator (30 kDa) and washed with Tris-HCl 50 mM pH 7.0 for buffer exchange. Protein concentration was determined using Pierce BCA Protein Assay Kit (Thermo Scientific).

### Thermal shift assay

**BDM76441** binding to MarR was assessed using a thermal shift assay. Briefly, in the wells of a 96-well PCR plate, purified His-MarR protein (7.5 μM), **BDM76441** (0–100 μM), and the fluorescent dye 1xSYPRO (Invitrogen) were mixed in 50 mM Tris-HCl buffer (pH 7.0) to a final volume 10 μl. Samples were then submitted to increasing temperatures (40 °C to 90 °C at a rate of 0.05 °C/s) using a LightCycler 480 II (Roche). SYPRO fluorescence was monitored using the Red640 filter (Excitation; 498 nm, Emission 640 nm), and the data were analyzed using the LightCycler Thermal Shift Analysis software (Roche) for Tm determination.

### Electrophoretic mobility shift assays

A 498-bp DNA fragment containing the *marRAB* promoter and both MarR binding sites (*marO*) was amplified from *E. coli* gDNA by PCR using primers RH957 and RH1130 (Table S6). Purified recombinant His-tagged MarR (5 μM) was preincubated with **BDM76441** (0–100 μM final concentration) in 8 μl (15 min, 25 °C). Two microliter of purified *marO* (final concentration 100 nM) was added and further incubated (15 min, 25 °C) to allow MarR-*marO* binding. Samples were then loaded on a DNA retardation gel (6%) (Invitrogen), and electrophoresis was performed at 150 V for 45 min in 0.5 X TBE running buffer. The gel was then stained in a 50 ml 0.5 X TBE bath with 5 μl of 10 mg/ml ethidium bromide for 1 h. DNA was visualized using E-BOX VX2 (Vilbert Lourmat) and quantified using Gel Analysis tool of ImageJ software. Percentage of unbound protein was calculated relative to the control lacking the MarR protein and plotted as a function of **BDM76441** concentration.

### Biochemical assay to determine NfsA nitroreductase activity

NfsA biochemical activity was determined by monitoring the metabolism of nitro-aromatic substrates and the NADPH cofactors using mass spectrometry. Biochemical assays were setup by mixing purified recombinant His-tagged NfsA (1 μM, final concentration), the nitro-aromatic compound (100 μM) and NADPH (200 μM) in 50 μl in Tris-HCl 50 mM pH7.0. Following incubation for the indicated times (37 °C), 10 μl of the reaction mixture was injected for mass spectrometry analysis. The UHPLC-MS used was a Dionex UltiMate 3000 system coupled to an LCQ Fleet ion trap mass spectrometer (Thermo Scientific). Sample separation was achieved using a reverse phase column Acquity UPLC peptide BEH C18 column (pore size 300 Å, particle size 1.7 μm, 2.1 × 150 mm, Waters, Milford, MA), using a mobile phase gradient of mobile phase A (water, 0.1% formic acid, v/v) to mobile phase B (acetonitrile, 0.1% formic acid, v/v) over 10 min (flow rate: 0.4 ml/min). To capture reactive intermediates of NfsA catalyzed metabolites, glutathione was added to the initial reaction mix (200 μM).

To evaluate NADPH consumption and NADP+ production an alternative UHPLC-MS method was adapted from literature (41). Briefly, similar to above, a reaction mixture containing NfsA (3 μM), NADPH (1 mM), and MTZ (1 mM) or NFZ (1 mM) was prepared in 50 μl, and samples injected at indicated times into an alternative UHPLC-MS method. Ten microliter of the reaction mix was injected on the same UHPLC-MS spectrometer as above using the same C18-column; however, the mobile phase consisted of a linear gradient from solvent A (triethylamine 100 mM, acetic acid 100 mM, 3% methanol, pH 6.5) to B (100% methanol) over 8 min.

### CV measurements

CV experiments were carried out on an IKA ElectraSyn 2.0 potentiostat using the following conditions: 0.5 M citrate buffer (pH 7.4)/dimethylformamide (3/2, v/v), Ag/Ag^+^ as a reference electrode, carbon glassy working electrode, and Pt counter electrode, with a scan rate of 100 mV/s. Deionized water was prepared with a Milli-Q water purification system to the resistivity of 17 MΩ cm. The solvents were degassed by bubbling the solutions with nitrogen for at least 20 min to eliminate interference from dissolved oxygen, and the respective solutions were filtered through a 200 nm Teflon filter prior to all measurements. Ferrocene was used as received (Alfa Aesar, 99%). Before each measurement, the platinum and carbon electrodes were rinsed with acetone and DI water and polished with an alumina slurry. The stock solutions of MTZ (4 mM) and nitrofurazone (4 mM) in the water–dimethylformamide mixture were prepared by mass (±0.001 mg). The dilute (2 mM) solutions of MTZ and nitrofurazone standardized against Fc/Fc+ (ferrocenium/ferrocene) redox couple were prepared by mixing the respective 4 mM solutions in 1:1 (v/v) ratio with the solution of ferrocene (4 mM, dissolved using an ultrasonic bath). The direction of the initial scan was negative in both cases. The pH control was measured with a Seven Compact S220 (Mettler Toledo) pH-meter with the combined glass electrode InLab Routine Pro-ISM at room temperature.

### EPR experiments

Continuous wave X-band measurements were recorded using an X-band Bruker E500 instrument (9.4 GHz, TE012 resonator). All continuous wave experiments were recorded at room temperature. Capillary (1 mm) sealed at one side was inserted in a 4 mm tube and then recoded with a modulated frequency of 100 KHz and modulation amplitude of 0.25 G. The hyperfine coupling constants for the MTZ anion radicals were determined using the Easyspin software (42).

### Computational details

All theoretical calculations were based on the DFT and were performed with the ORCA program package (43). Full geometry optimizations as well as electronic structure calculations were undertaken for all systems using the hybrid functional B3LYP (44, 45) in combination with the TZV/P (46) basis set for all atoms and by taking advantage of the resolution of the identity approximation in the Split-RI-J variant (47) with the appropriate Coulomb fitting sets (48). Increased integration grids (Grid4 and GridX4 in ORCA convention) and tight self-consistent-field convergence criteria were used in the calculations. To ensure that the resulting structures converged to a local minimum on the potential energy surface, numerical frequency calculations were performed and resulted in only positive normal vibrations. Solvent effects were accounted for according to the experimental conditions. For that purpose, we used water as a solvent (ε = 80) within the framework of the conductor like screening (COSMO) dielectric continuum approach (49). The Gibbs free energies were computed from the optimized structures as a sum of electronic energy, solvation, and thermal corrections to the free energy. Redox potentials were obtained from the calculated free energy change between oxidized and reduced species in solution. They are relative potentials referenced to standard hydrogen electrode, and as such, a value of 4.28 eV was subtracted to make direct comparisons to experimental data (50, 51). An additional pH∗0.0591 V term can be added to the potential to account for the pH conditions. EPR parameters were obtained from additional single-point calculations using the EPR-II basis set (52) and the hybrid functional B3LYP. Molecular orbitals and spin densities were generated using the orca_plot utility program. Optimized geometries as well as electronic structures were visualized with the Chemcraft (www.chemcraftprog.com) program.

## Data availability

Data can be shared on request to the corresponding author.

## Supporting information

This article contains supporting information.

## Conflict of interest

The authors declare no conflict of interest with the contents of this article.
