# Supplementary material for: Small molecule MarR modulators potentiate metronidazole antibiotic activity in aerobic E. coli by inducing activation by the nitroreductase NfsA
Source: J Biol Chem. 2024 May 31;300(7):107431. doi: 10.1016/j.jbc.2024.107431 (PMC11259696; doi:10.1016/j.jbc.2024.107431)
Supplement: Supporting Information [file mmc1.docx]

**Supporting Information**

**Figures and Tables**

**Small molecule MarR modulators potentiate metronidazole antibiotic activity in aerobic *E. coli* by inducing activation by the nitroreductase NfsA**

Thibault Caradec^1^, Coline Plé^1^, Giuseppe Sicoli^2^, Ravil Petrov^1^, Elizabeth Pradel^1^, Cecilia Sobieski^1^, Rudy Antoine^1^, Maylis Orio^3^, Adrien Herledan^4^, Nicolas Willand^4^, Ruben C. Hartkoorn^1^#

^1^ Univ. Lille, CNRS, Inserm, CHU Lille, Institut Pasteur Lille, U1019 - UMR 9017 - CIIL - Center for Infection and Immunity of Lille, F-59000 Lille, France

^2^ CNRS UMR 8516, Univ. Lille, LASIRE – Laboratory of Advanced Spectroscopy on Interactions, Reactivity and Environment, C4 Building, Avenue Paul Langevin, F–59655 Villeneuve d’Ascq, France.

^3^ Aix Marseille Univ., CNRS, Centrale Marseille, iSm2, Marseille 13397, France.

^4^ Univ. Lille, Inserm, Institut Pasteur de Lille, U1177 - Drugs and Molecules for Living Systems, F-59000, Lille, France.

# Corresponding Author: ruben.hartkoorn@inserm.fr


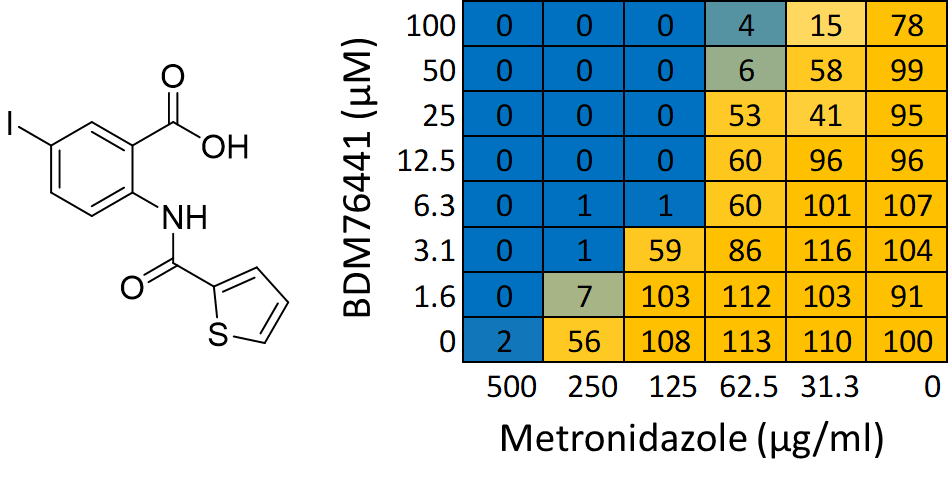


**Figure S1: Solid media checkerboard assay:** *E. coli* ∆*tolC* viability was evaluated on solid media (CAMHB with agar) spiked with different concentrations of **BDM76441** (structure in figure) and MTZ. Following overnight culture at 37°C, bacterial growth was quantified by the addition of resazurin, and indicated as a percentage resorufin fluorescence compared to untreated bacteria. Values in the checkerboard assay are the mean percentage of resazurin turnover measured in two independent experiments.


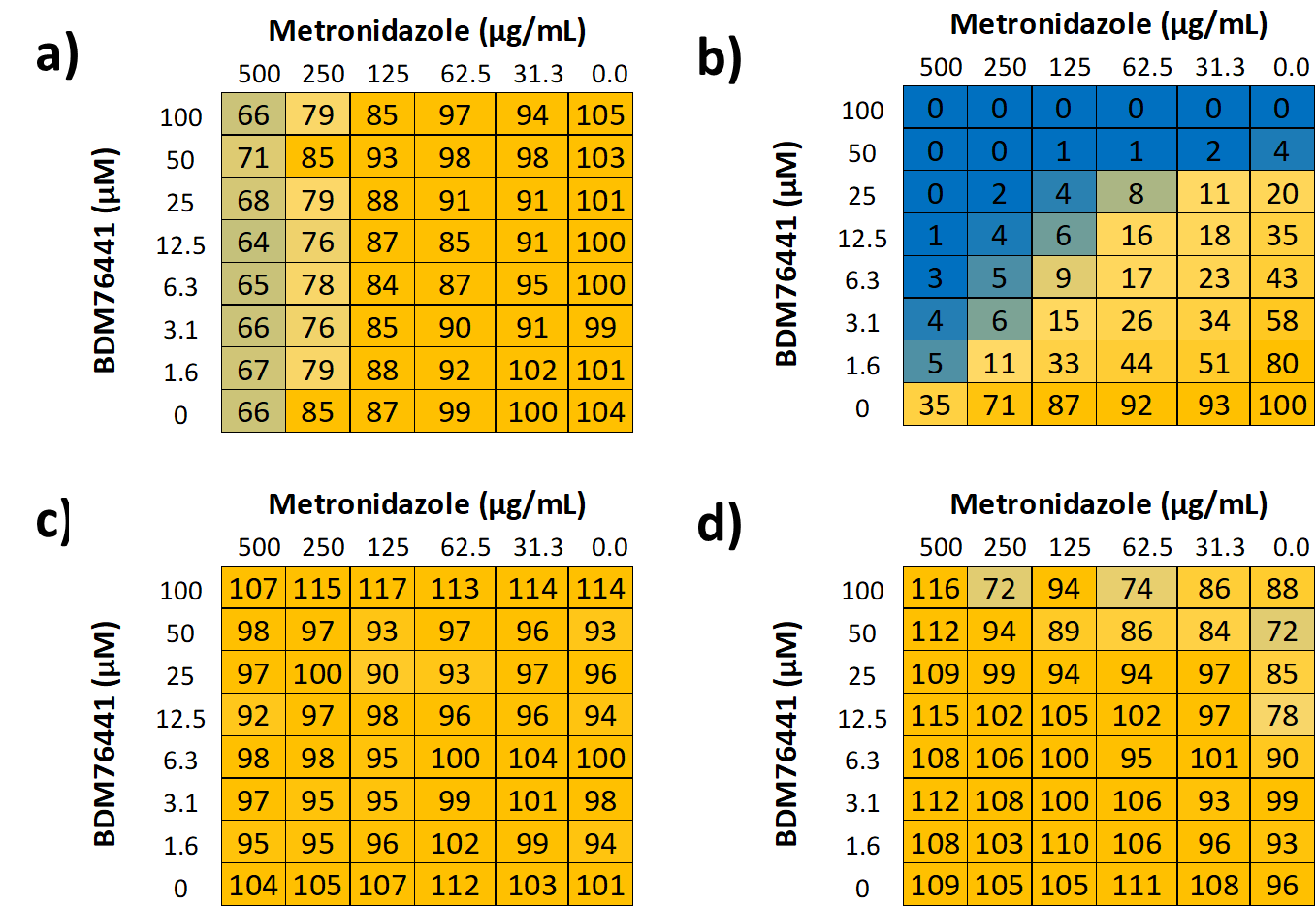


**Figure S2: Liquid culture checkerboard assay:** The viability of *E. coli* strains **a**) BW25113 WT, **b**) *E. coli* BW25113 ∆*tolC*, **c**) CFT073, **d**) Nissle 1917 was evaluated in CAMHB spiked with different concentrations of **BDM76441** and MTZ. Following 5 h culture at 37°C, bacterial growth was quantified by the addition of resazurin, and indicated as a percentage resorufin fluorescence compared to untreated bacteria. Values in the checkerboard assay are the mean percentage of resazurin turnover measured in two independent experiments.

**
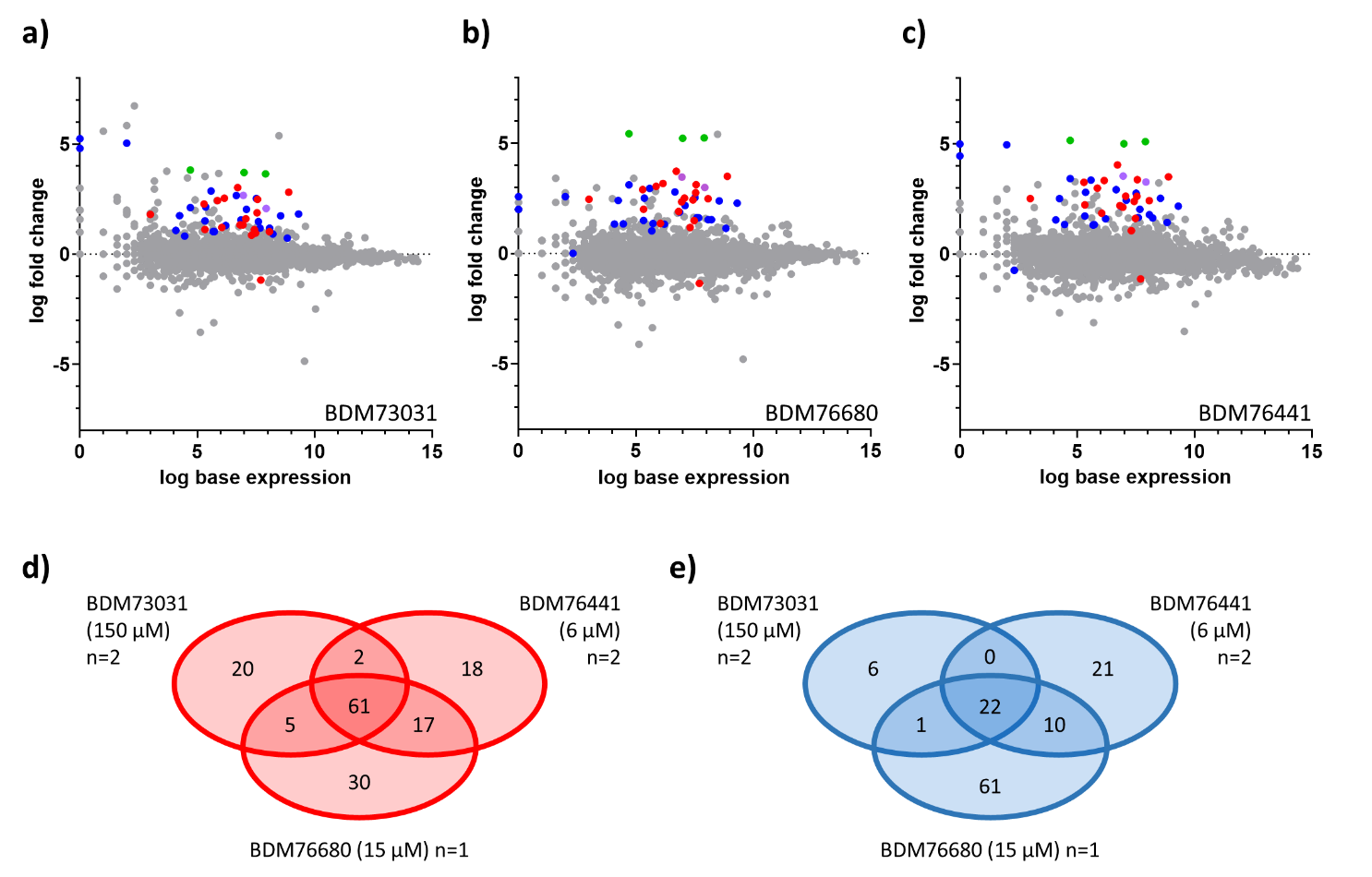
**

**Figure S3:** MA plots (**a-c**) representation of the transcriptional stress response of *E. coli* ∆*tolC* under aerobic conditions to 1 h exposure of **a**) 150 µM **BDM73031**, **b**) 15 µM **BDM76680** or **c**) 6 µM **BDM76441**. Coloured dots represent the *marRAB* genes (green), the *nfsA* and *B* nitro-reductase genes (purple), genes of the MarA regulon (red), and genes of the glutathione/ glucuronide pathways (blue). Venn-diagrams (**d-e**) summarising the commonality of **d**) upregulated and **e**) downregulated genes following exposure to the three MTZ boosters in *E. coli* Δ*tolC*.

**
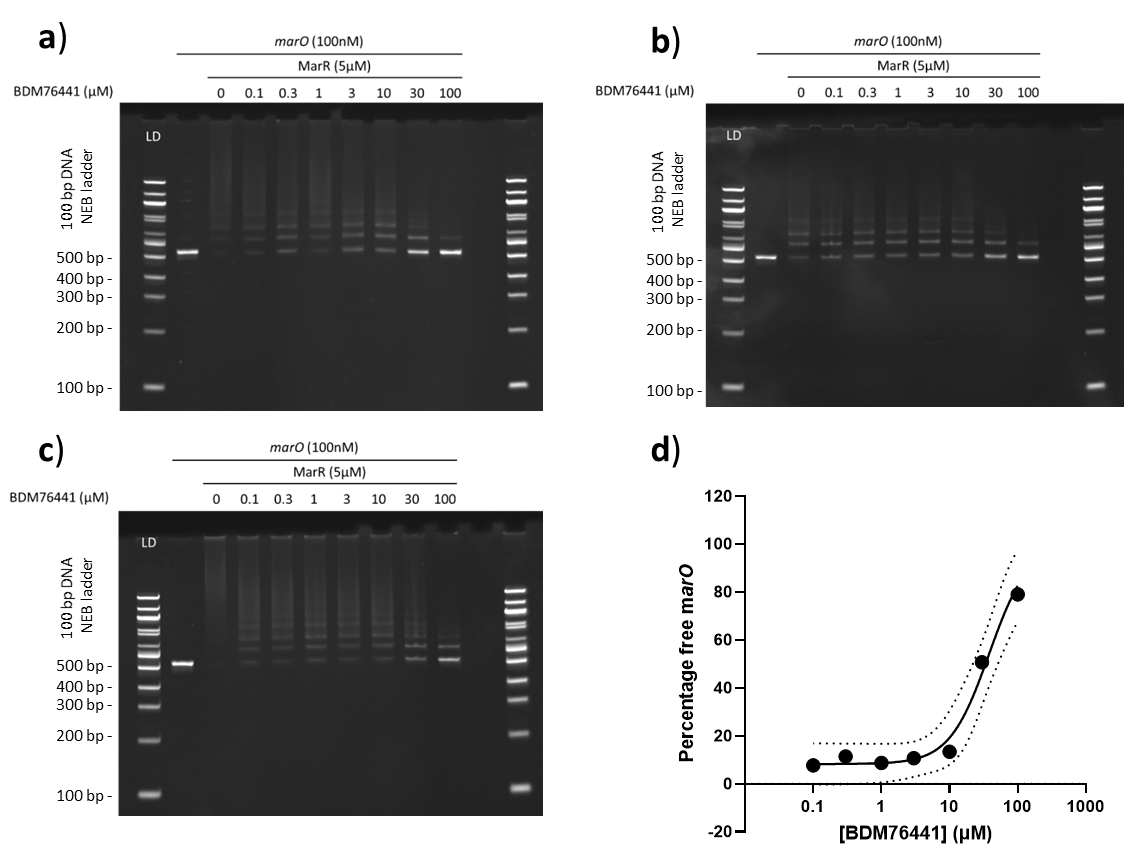
Figure S4:** **a-c**) Source EMSA gel images of the three independent biological replicate EMSA agarose gels looking at the migration of the *marRAB* promoter region (*marO*) in the presence of purified recombinant MarR and a dose range of **BDM76441**. Gel image **S4c**, is the source EMSA image for the manuscript **Fig 3b** (reuse) LD: NEB 100-bp DNA Ladder. **d**) Summary graph plot of the three EMSA gels showing the free *marO* percentage as a factor of **BDM76441** concentration. The intensity of the *marO* band was quantified using image J (maximum intensity across band). Graph shows the mean and 95% CI for the three gels.


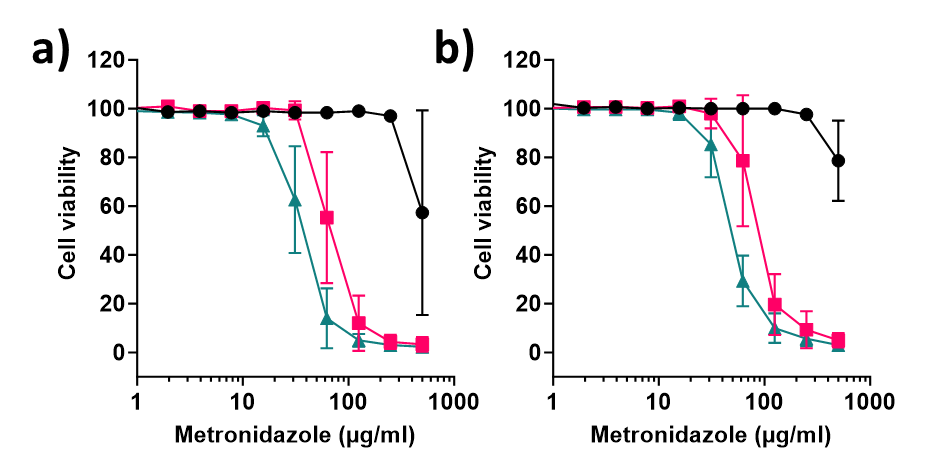


**Figure S5**: MTZ antibiotic activity on **a**) *E. coli* Δ*tolC* and **b**) *E. coli ∆tolC* *marR*-C80S, in the absence (black lines) or presence of 2 µM (red) or 6 µM **BDM76441** (green). Data are the mean ± SD of at least two independent experiments.


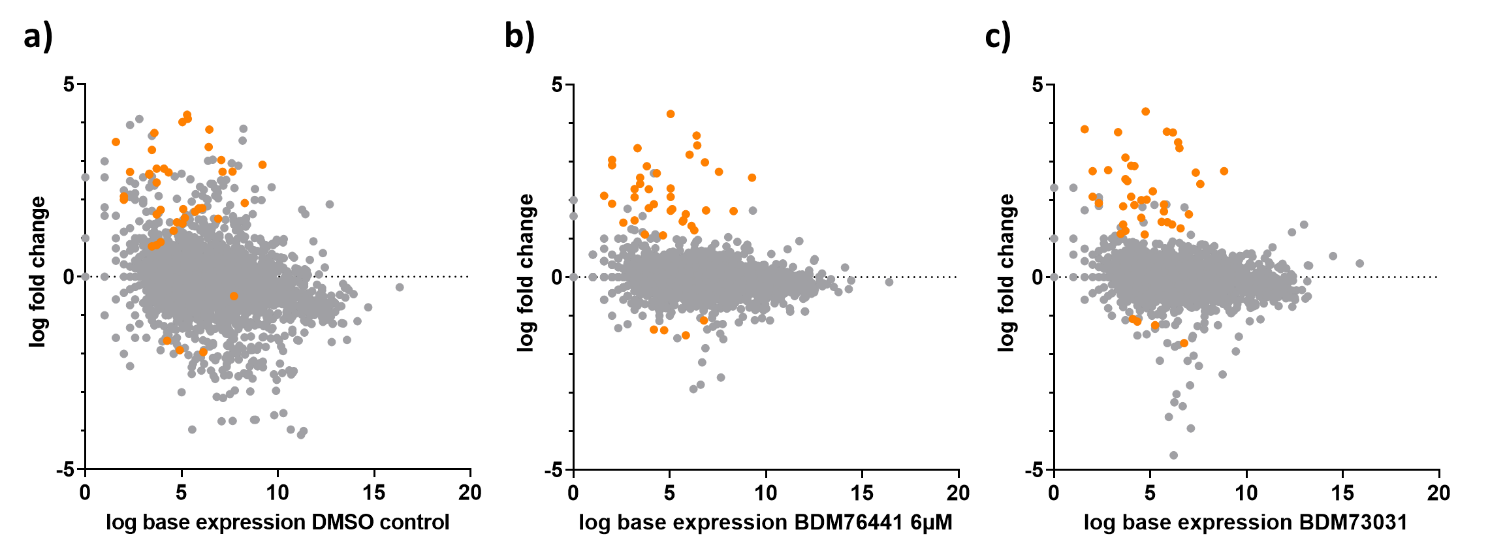


**Figure S6**: MA plot showing the impact on *E. coli* ∆*tolC* gene expression of a high dose of MTZ (2 mg/ml) **a**), or a low dose of MTZ (0.1 mg/ml) in combination with either 6 µM **BDM76441** **b**), or 150 µM **BDM73031** **c**). Genes belonging to the LexA regulon are indicated in orange.


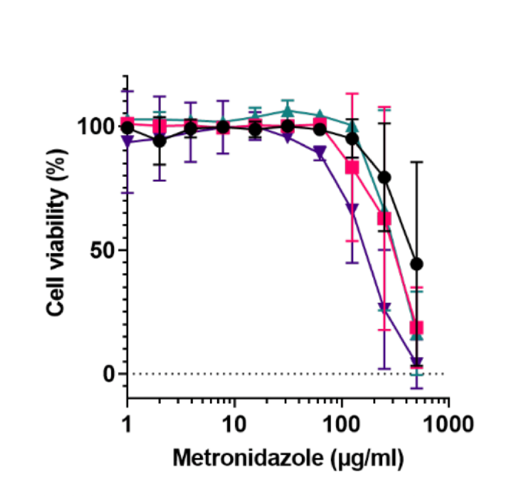


**Figure S7**: MTZ antibiotic activity on *E. coli* Δ*tolC* containing pBAD30::nfsB, in the absence of arabinose (black); or with 0.1 % (pink), 0.5 % (green) or 5 % arabinose (purple). Data are the mean ± SD of at least three independent experiments.


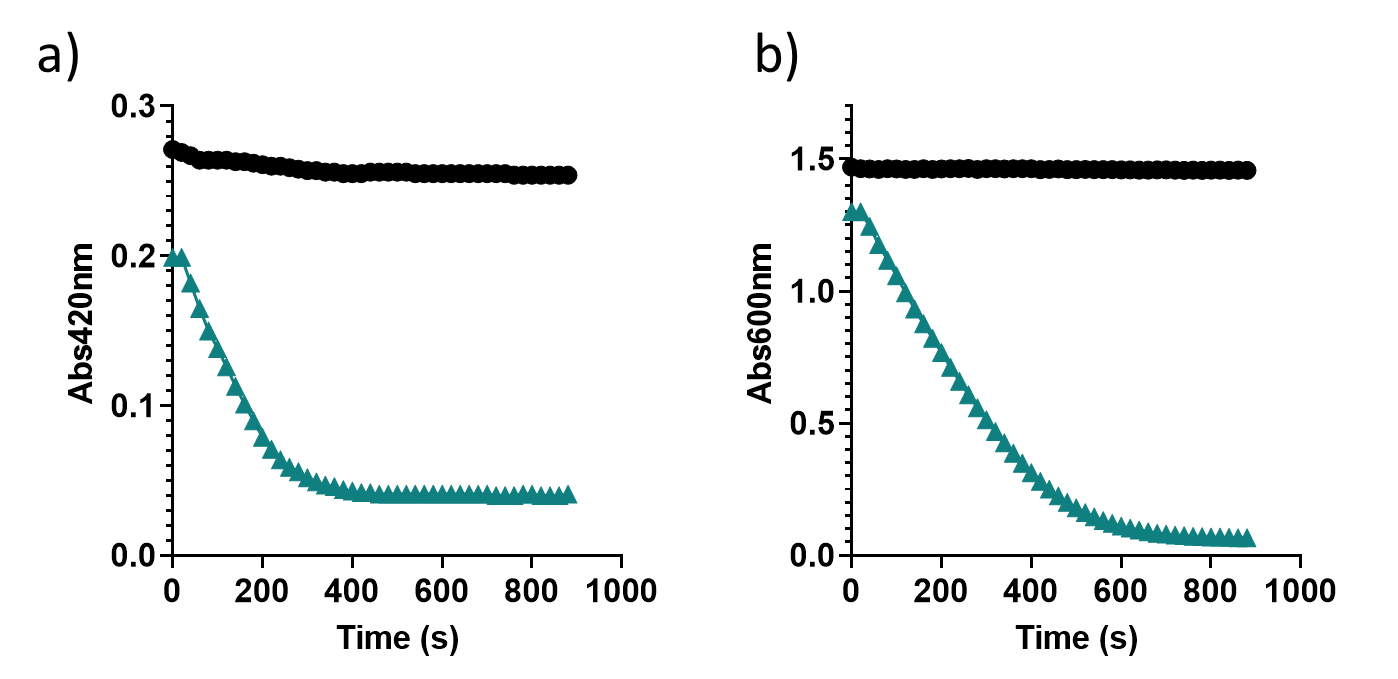


**Figure S8**: Reduction of 1-electron acceptor potassium ferricyanide **a**), or DCPIP **b**) by nitroreductase NfsA in the absence (black) or presence (green) of NADPH. Reduction was followed by absorbance at 420 nm for potassium ferricyanide and 600 nm for DCPIP.


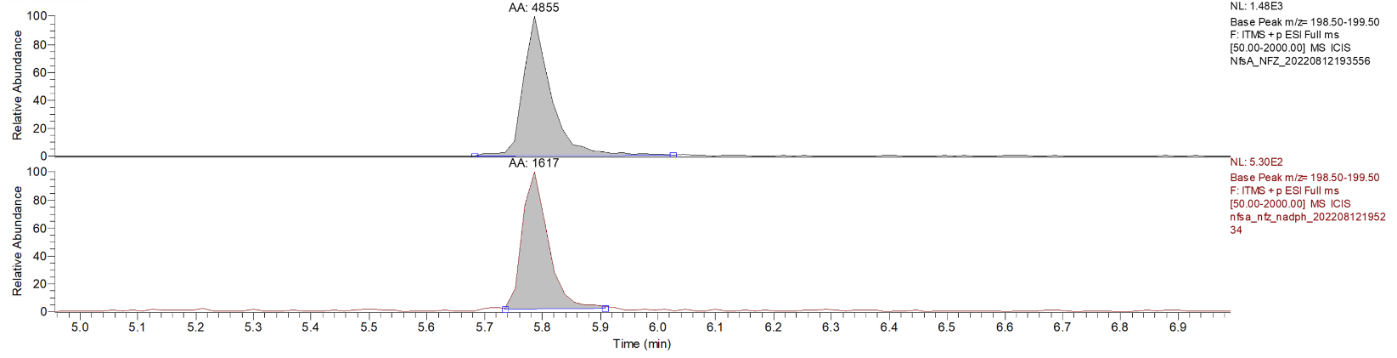


**Figure S9**: NFZ quantification in a reaction mix containing 1 µM NfsA, 100 µM NFZ, without (top) or with (bottom) 200 µM NADPH in 50 µL Tris HCl 50 mM pH 7.0, after incubation for 30 min at 37 °C. Area (AA) is evaluated for the peak of the [M+H]^+^ signal of NFZ (m/z = 199).


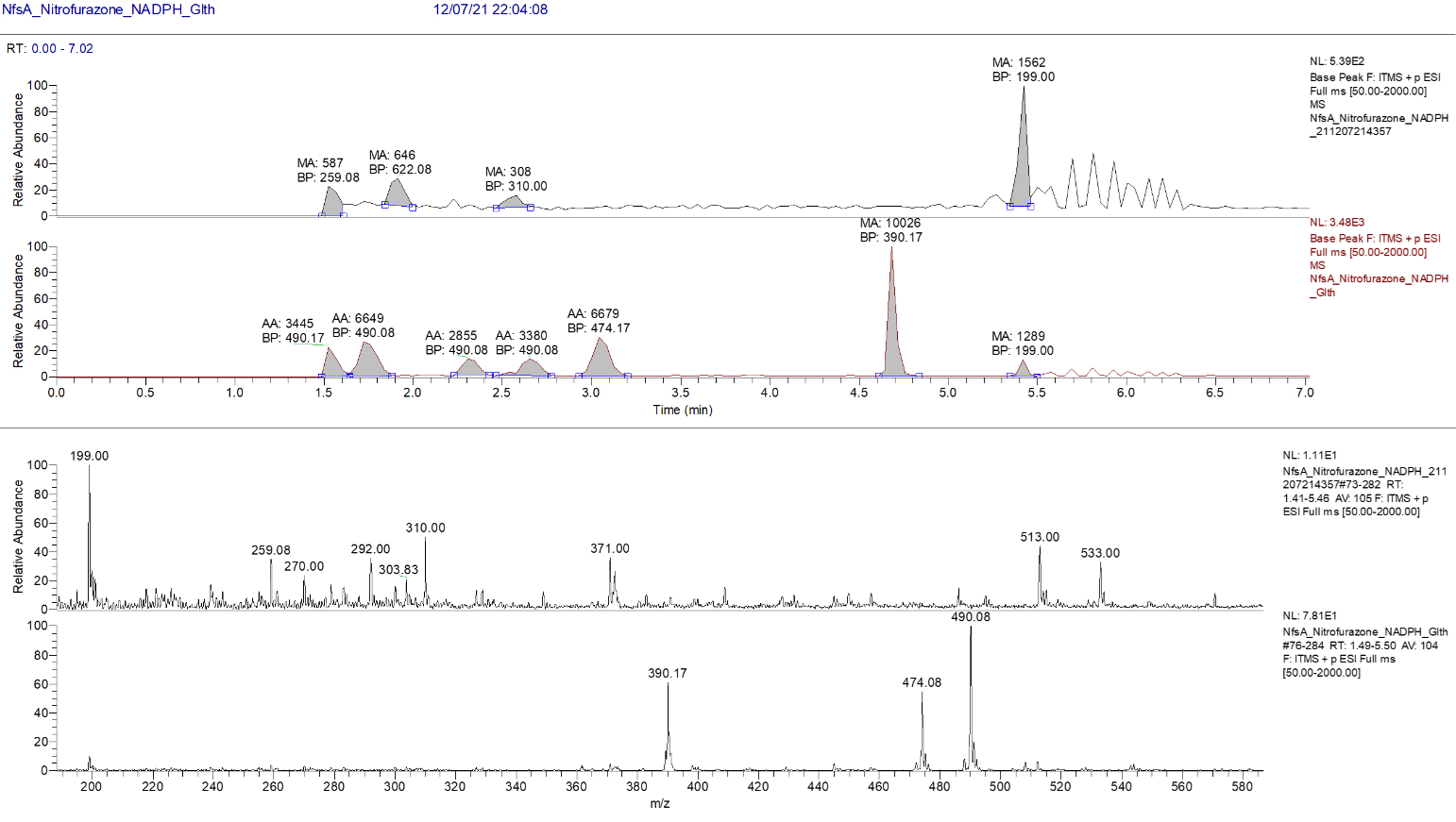


**Figure S10**: Mass spectrometry analysis of a mix of 1 µM NfsA, 100 µM NFZ and 100 µM glutathione without (top) and with (bottom) 200 µM NADPH in 50 µL Tris HCl 50 mM pH7.0, after incubation for 30 min at 37°C.


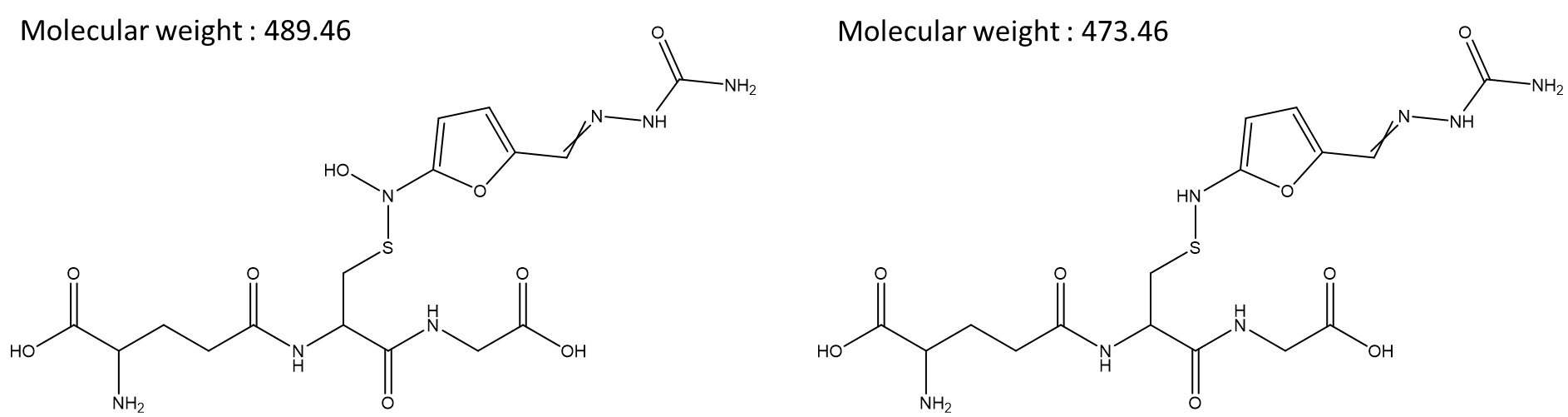


**Figure S11**: Proposed structures and calculated molecular weights of NFZ-glutathione conjugates.


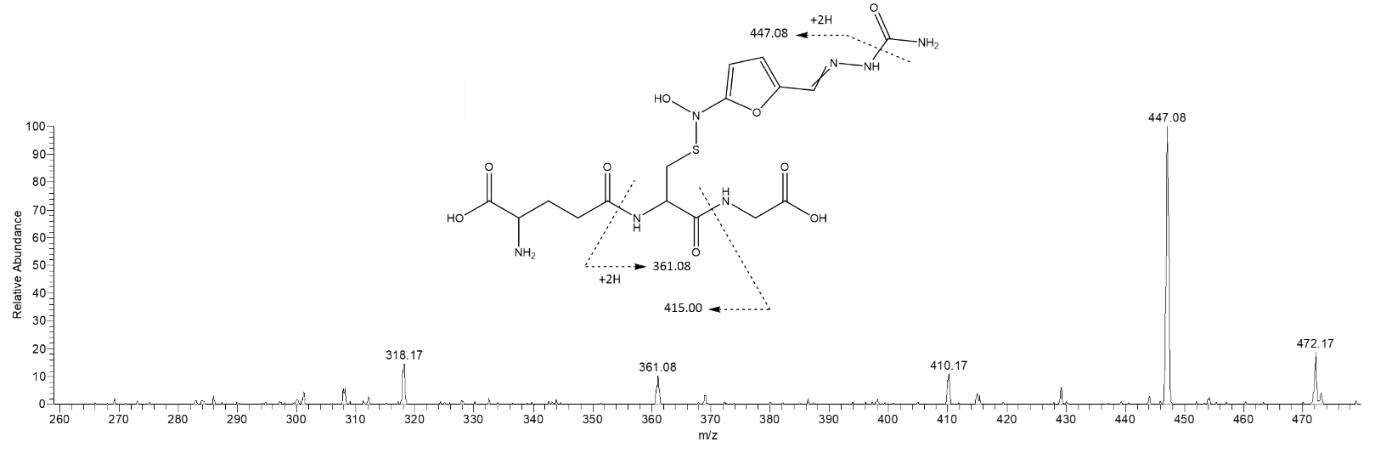


**Figure S12**: Mass spectrometry fragmentation of predicted NFZ-glutathione conjugate with m/z 490 to daughter ions. The potential allocation of some of the daughter ions is shown in the inlay.


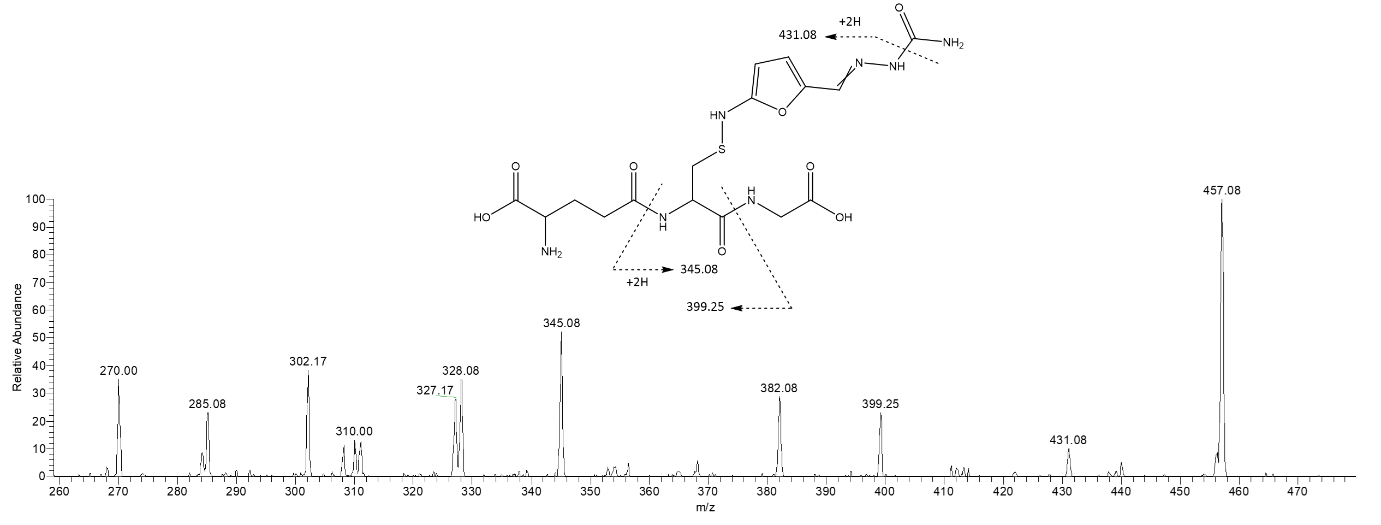


**Figure S13** : Mass spectrometry fragmentation of second predicted NFZ-glutathione conjugate with m/z 474 to daughter ions. The potential allocation of some of the daughter ions is shown in the inlay.

**Figure S14**: Nitroaromatics quantification in a reaction mix containing NfsA, NFZ or MTZ, without or with NADPH. Results are expressed as percentage of remaining nitroaromatic compound after 30 min incubation as detected by LC-MS. Data are the mean ± SD of three independent experiments.


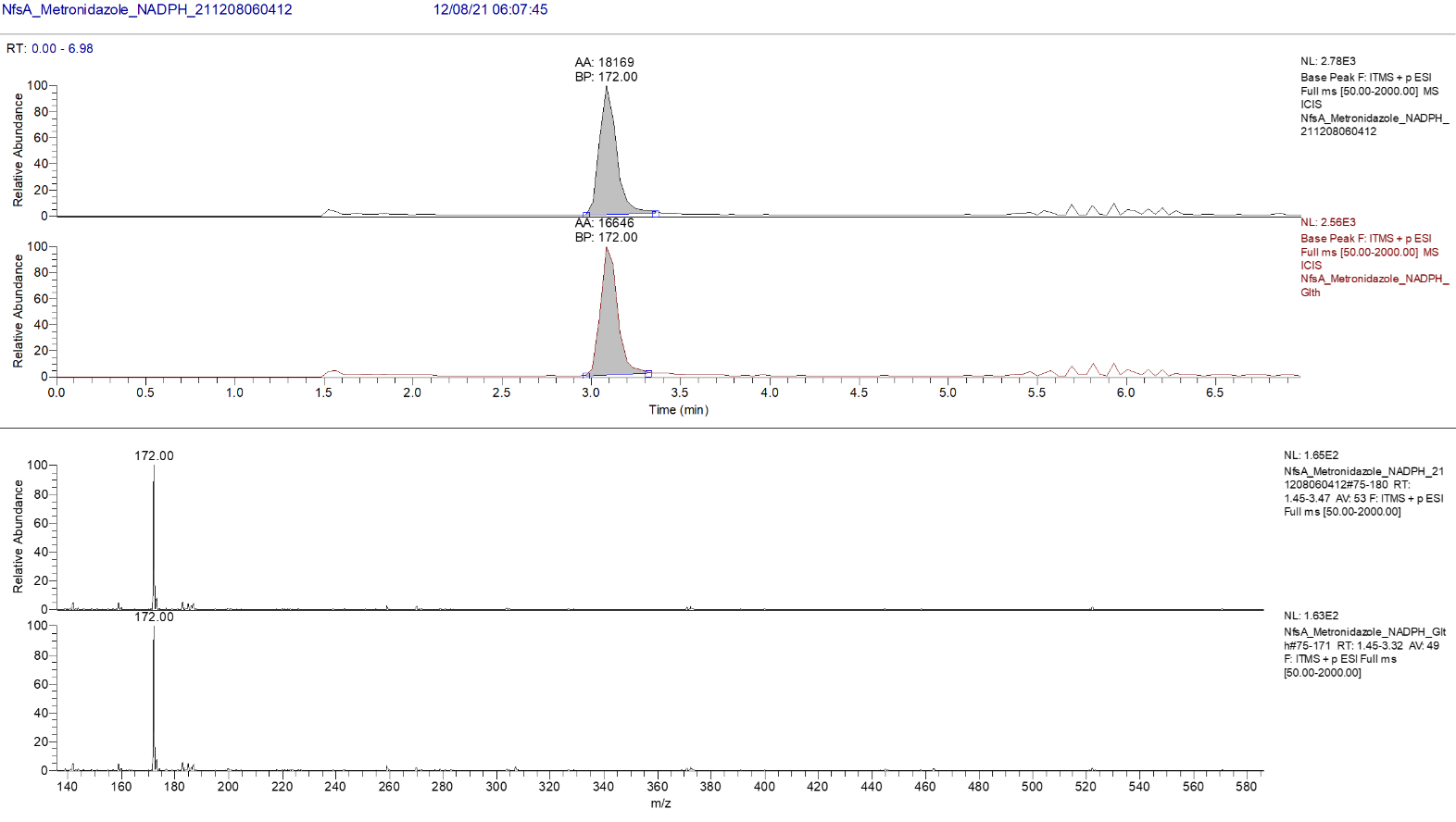


**Figure S15**: Mass spectrometry analysis of a mix of 1 µM NfsA, 100 µM metronidazole and 100 µM glutathione without (top) and with (bottom) 200 µM NADPH in 50 µL Tris HCl 50 mM pH7.0, after incubation for 30 min at 37°C.


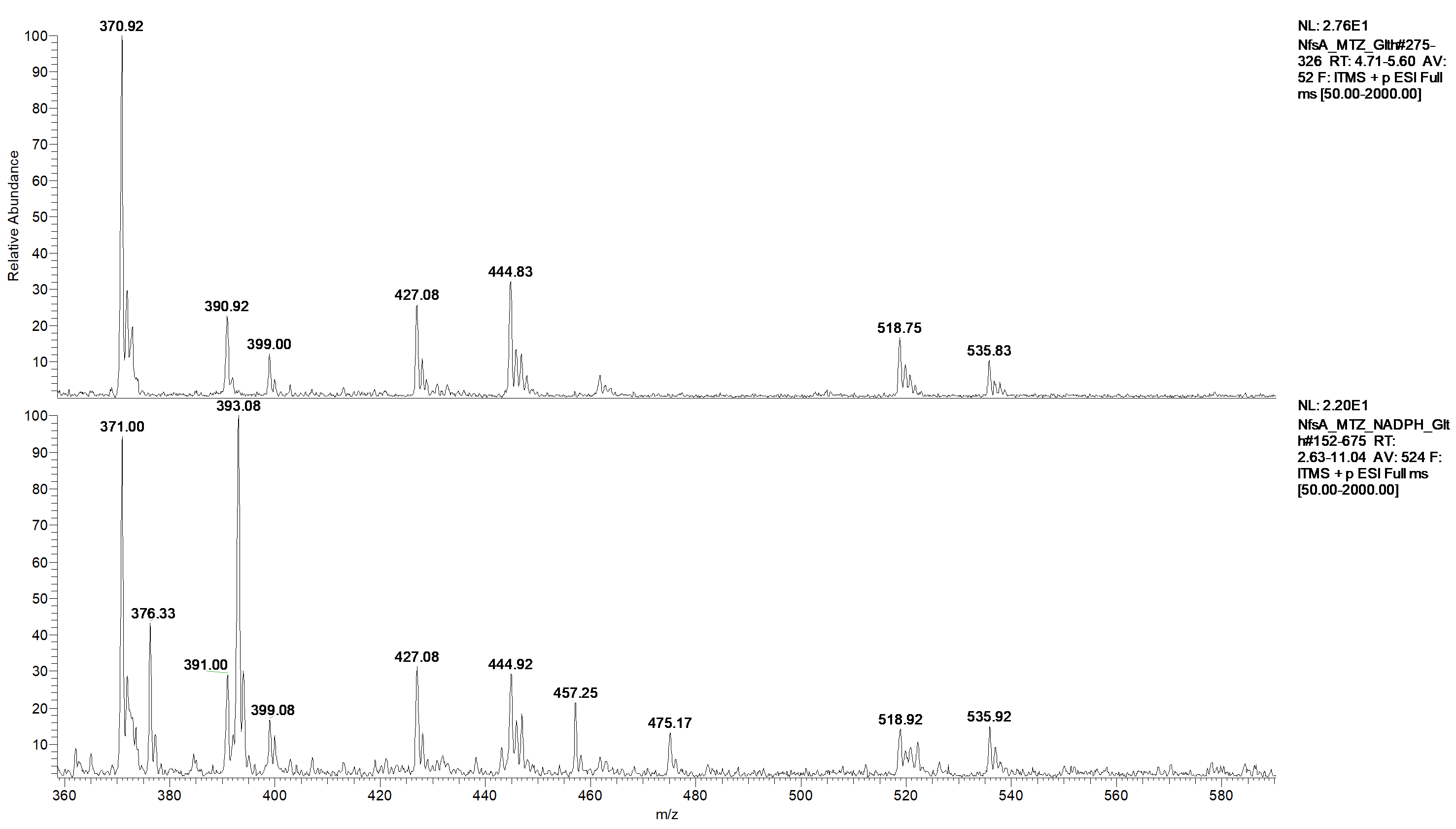


**Figure S16**: Mass spectrometry analysis of a mix of 1 µM NfsA, 100 µM metronidazole and 100 µM glutathione without (top) and with (bottom) 200 µM NADPH in 50 µL Tris HCl 50 mM pH7.0, after incubation for 30 min at 37°C. Expected mass for metronidazole glutathione adducts are m/z 447 and 473.


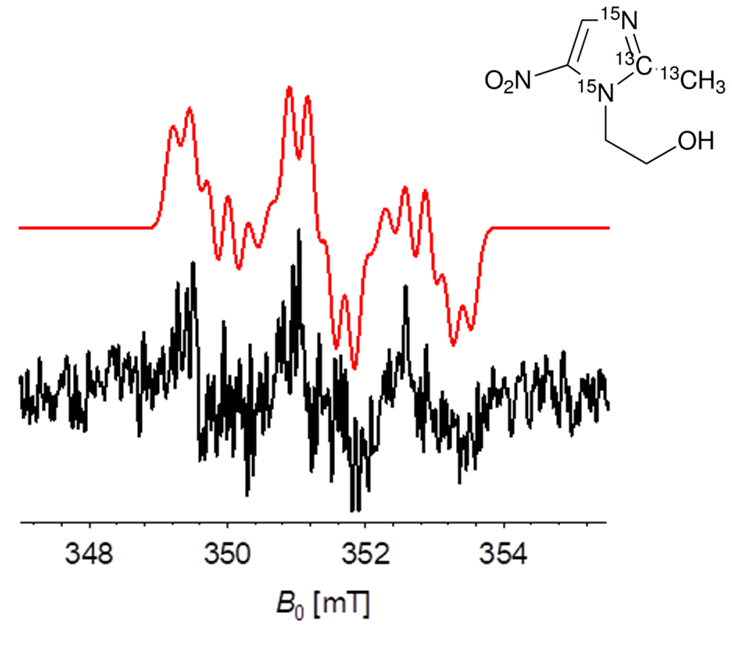


**Figure S17**: CW EPR spectrum (bottom) and spectra simulation (top) for ^14^N – ^13^C labelled MTZ radical anion.

**Table S1**: **BDM76441** mediated potentiation of the antibiotic activity of some commercially available nitroimidazoles and nitrofuranes in *E. coli ∆tolC* under aerobic conditions. Bacterial viability was determined using the resazurine reduction assay, and values and ranges are the results of two independent experiments.

| **Antibiotic** | **Structure** | **MIC_90_ against E. coli ∆tolC under aerobic conditions (µg/mL)** | | | **Potentiation** |
| --- | --- | --- | --- | --- | --- |
|  |  | **Alone** | **With 4 µM BDM76441** | **With 16 µM BDM76441** |  |
| Metronidazole |  | >500 | 250 | 62.5-125 | Yes |
| Dimetridazole |  | >500 | >500 | >500 | ? |
| Metronidazole acetic acid (STK298623) |  | >500 | >500 | >500 | ? |
| Secnidazole |  | >500 | >500 | >500 | ? |
| Ornidazole |  | >500 | >500 | >500 | ? |
| Ternidazole |  | >500 | >500 | >500 | ? |
| STK183723 |  | 500 | 62.5 | 62.5 | Yes |
| STK251667 |  | 250 | 125 | 62.5 | Yes |
| STK238676 |  | 500 | 250 | 125 | Yes |
| STK317892 |  | >500 | >500 | >500 | ? |
| STK261833 |  | >500 | >500 | >500 | ? |
| Benznidazole |  | 62.5 | 62.5 | 62.5 | No |
| Nimorazole |  | >500 | >500 | >500 | ? |
| Ronidazole |  | >500-250 | 250 | 250 | No |
| Nitrofurazone |  | 15.6 | 15.6 | 15.6 | No |

**Table S2:** The transcriptional stress response of *E. coli ∆tolC* under aerobic conditions to 1 h exposure of 150 µM **BDM73031** (n=2), 15 µM **BDM76680** and 6 µM **BDM76441** (n=2). Values are expressed as log_2_(rpkm) fold change in expression compared to the DMSO exposed control. Y: yes, ind: indirectly through small RNA micF.

|  |  |  |  |  |  | **Change in Log(2)_RPKM** | | | | |
| --- | --- | --- | --- | --- | --- | --- | --- | --- | --- | --- |
| **locus_tag BW25113_** | **old_locus_tag BW25113_** | **Gene** | **MarA regulon** | **Thio/sulfate, cysteine, glutathione, glucuronide pathways** | **Gene Product** | **BDM73031 vs DMSO (n=1)** | **BDM73031 vs DMSO (n=2)** | **BDM76680 vs DMSO (n=1)** | **BDM76441 _vs DMSO (n=1)** | **BDM76441 vs DMSO (n=2)** |
|  |  |  |  |  |  |  |  |  |  |  |
| RS00075 | 15 | *dnaJ* |  |  | molecular chaperone DnaJ | 1.16 | 1.4 | 0.45 | 1.12 | 1.21 |
| RS00355 | 71 | *leuD* |  |  | 3-isopropylmalate dehydratase small subunit | 1.58 | 0.58 | 2 | 2.32 | 1.12 |
| RS00620 | 124 | *gcd* |  |  | quinoprotein glucose dehydrogenase | 0.97 | 1.75 | 1.21 | 1.16 | 1.53 |
| RS00635 | 127 | *yadG* | Y |  | ABC transporter ATP-binding protein | 2.49 | 2.77 | 3.13 | 3.38 | 3.62 |
| RS00640 | 128 | *yadH* | Y |  | ABC transporter permease | 2.28 | 2.61 | 2.9 | 3.26 | 3.52 |
| RS00645 | 129 | *yadI* | Y |  | PTS sugar transporter subunit IIA | 1.81 | 1.57 | 2.46 | 2.52 | 2.14 |
| RS01065 | 212 | *gloB* |  |  | hydroxyacylglutathione hydrolase | 0.84 | 1.29 | 1.48 | 1.58 | 1.97 |
| RS01905 | 369 | *hemB* | Y |  | porphobilinogen synthase | 1.88 | 1.47 | 2.76 | 2.62 | 2.34 |
| RS02385 | 462 | *acrB* | Y |  | efflux RND transporter permease AcrB | 1.12 | 0.82 | 2.44 | 2.39 | 2.42 |
| RS02390 | 463 | *acrA* | Y |  | RND efflux transporter periplasmic adaptor subunit AcrA | 1.02 | 1.04 | 2.49 | 2.43 | 2.61 |
| RS02475 | 479 | *fsr* |  |  | fosmidomycin MFS transporter | 1.69 | 2.12 | 1.42 | 1.08 | 1.05 |
| RS02855 | 553 | *ompC* |  |  | porin OmpC | 4.86 | 4.84 | 4.8 | 3.51 | 3.67 |
| RS02875 | 557 | *bor* |  |  | serum resistance lipoprotein Bor | 2.49 | 4.42 | 1.92 | 2.27 | 3.29 |
| RS02985 | 576 | *pheP* |  |  | phenylalanine transporter | 1.24 | 1.58 | 1.89 | 1.7 | 1.87 |
| RS02995 | 578 | *nfsB* | Y |  | oxygen-insensitive NAD(P)H nitroreductase | 2.07 | 2.03 | 3 | 3.28 | 3.15 |
| RS03140 | 603 | *ybdO* |  |  | LysR family transcriptional regulator | 1.81 | 3.46 | 2.58 | 1.91 | 3.17 |
| RS03960 | 762 | *acrZ* | Y |  | multidrug efflux pump accessory protein AcrZ | 1.33 | 1.07 | 2.35 | 2.13 | 1.98 |
| RS04115 | 792 | *ybhR* |  |  | ABC transporter permease | 1.35 | 0.98 | 1.06 | 1.58 | 1.02 |
| RS04120 | 793 | *ybhS* |  |  | ABC transporter permease | 1.49 | 1.02 | 1.09 | 1.72 | 1.01 |
| RS04125 | 794 | *ybhF* |  |  | ATP-binding cassette domain-containing protein | 1.4 | 0.96 | 1.01 | 1.64 | 1.09 |
| RS04300 | 828 | *iaaA* |  |  | beta-aspartyl-peptidase | 1.62 | 2.1 | 1.58 | 1.98 | 1.18 |
| RS04305 | 829 | *gsiA* |  | Y | glutathione ABC transporter ATP-binding protein GsiA | 1.51 | 2.11 | 1.5 | 1.73 | 1.08 |
| RS04310 | 830 | *gsiB* |  | Y | glutathione ABC transporter substrate-binding protein GsiB | 1.31 | 1.93 | 1.33 | 1.61 | 1.06 |
| RS04315 | 831 | *gsiC* |  | Y | glutathione ABC transporter permease GsiC | 1.03 | 1.65 | 1.03 | 1.33 | 1.1 |
| RS04375 | 843 | *ybjH* |  |  | hypothetical protein | 2.96 | 2.29 | 1.29 | 1.47 | 1.14 |
| RS04410 | 850 | *ybjC* | Y |  | DUF1418 family protein | 2.81 | 2.39 | 3.51 | 3.51 | 3.23 |
| RS04415 | 851 | *nfsA* | Y |  | nitroreductase NfsA | 2.67 | 2.34 | 3.47 | 3.55 | 3.27 |
| RS04420 | 852 | *rimK* | Y |  | 30S ribosomal protein S6--L-glutamate ligase | 2.54 | 2.26 | 3.18 | 3.34 | 3.15 |
| RS04520 | 871 | *poxB* | Y |  | ubiquinone-dependent pyruvate dehydrogenase | 1.12 | 2.64 | 2.01 | 2.24 | 2.73 |
| RS04820 | 929 | *ompF* | ind |  | porin OmpF | 1.76 | 2.12 | 1.85 | 1.8 | 1.99 |
| RS05490 | 1053 | *mdtG* |  |  | multidrug efflux MFS transporter MdtG | 1.91 | 2 | 2.32 | 1.46 | 1.22 |
| RS05495 | 1054 | *lpxL* |  |  | kdo(2)-lipid IV(A) lauroyltransferase | 2 | 1.92 | 2.44 | 2.53 | 2.28 |
| RS06675 | 1276 | *acnA* | Y |  | aconitate hydratase AcnA | 1.21 | 2.17 | 1.36 | 1.86 | 2.58 |
| RS06740 | 1287 | *yciW* |  |  | CMD domain-containing protein | 1.99 | 4.26 | 2.33 | 2.75 | 2.85 |
| RS07620 | 1451 | *yncD* |  |  | TonB-dependent receptor | 1.91 | 1.88 | 2.37 | 2.23 | 1.97 |
| RS08020 | 1530 | *marR* | Y |  | multiple antibiotic resistance transcriptional regulator MarR | 3.71 | 3.42 | 5.24 | 5.01 | 5.16 |
| RS08025 | 1531 | *marA* | Y |  | MDR efflux pump AcrAB transcriptional activator MarA | 3.65 | 2.89 | 5.26 | 5.11 | 5.02 |
| RS08030 | 1532 | *marB* | Y |  | multiple antibiotic resistance protein MarB | 3.83 | 2.78 | 5.45 | 5.16 | 4.85 |
| RS08450 | 1615 | *uidC* |  | Y | glucuronide uptake porin UidC | 4.81 | 6.23 | 2 | 4.46 | 5.17 |
| RS08455 | 1616 | *uidB* |  | Y | glucuronide transporter | 5.25 | 6.85 | 2.58 | 5 | 6.49 |
| RS08460 | 1617 | *uidA* |  | Y | beta-glucuronidase | 5.04 | 6.47 | 2.58 | 4.97 | 6.39 |
| RS08505 | 1625 | *ydgT* |  |  | transcription modulator YdgT | 1.1 | 0.87 | 1.38 | 1.97 | 1.87 |
| RS09045 | 1729 | *tcyP* |  | Y | cystine/sulfocysteine:cation symporter | 1.48 | 2.54 | 1.63 | 1.66 | 1.09 |
| RS09710 | 1852 | *zwf* | Y |  | glucose-6-phosphate dehydrogenase | 0.96 | 1.34 | 1.49 | 1.62 | 2.21 |
| RS09950 | 1902 | *ftnB* |  |  | non-heme ferritin-like protein | 1.44 | 1.42 | 2.25 | 1.93 | 1.91 |
| RS09970 | 1905 | *ftnA* |  |  | non-heme ferritin | 0.69 | 1.26 | 1.08 | 1.28 | 1.46 |
| RS10035 | 1917 | *yecC* |  | Y | L-cystine ABC transporter ATP-binding protein YecC | 0.83 | 1.34 | 1.35 | 1.35 | 1.44 |
| RS10040 | 1918 | *tcyL* |  | Y | cystine ABC transporter permease | 1.08 | 1.61 | 1.34 | 1.56 | 1.52 |
| RS10045 | 1919 | *dcyD* |  | Y | D-cysteine desulfhydrase | 1.03 | 1.58 | 1.36 | 1.34 | 1.52 |
| RS10235 | 1959 | *yedA* |  |  | drug/metabolite exporter YedA | 3 | 3 | 2.32 | 2 | 2.32 |
| RS10375 | 1987 | *cbl* |  | Y | HTH-type transcriptional regulator Cbl | 1.75 | 3.55 | 2.41 | 2.52 | 2.32 |
| RS10515 | 2012 | *yeeD* |  | Y | sulfurtransferase TusA | 1.82 | 1.77 | 2.29 | 2.18 | 1.11 |
| RS10520 | 2013 | *yeeE* |  | Y | transport protein YeeE | 2.53 | 4.23 | 2.54 | 2.77 | 2.4 |
| RS10650 | 2039 | *rfbA* |  |  | glucose-1-phosphate thymidylyltransferase RfbA | 0.59 | 1.02 | 1.09 | 1.29 | 2.05 |
| RS11345 | 2173 | *yeiR* |  |  | zinc-binding GTPase YeiR | 2.43 | 2.72 | 2.82 | 2.65 | 2.43 |
| RS11350 | 2174 | *lpxT* |  |  | Kdo(2)-lipid A phosphotransferase | 1.89 | 2.33 | 2.17 | 2.26 | 1.86 |
| RS11685 | 2237 | *inaA* | Y |  | lipopolysaccharide kinase InaA | 2.44 | 3.12 | 3.05 | 3 | 3.27 |
| RS11755 | 2250 | *yfaZ* |  |  | YfaZ family protein | 1.58 | 2 | 1.58 | 1.58 | 1.74 |
| RS12635 | 2422 | *cysA* |  | Y | sulfate/thiosulfate ABC transporter ATP-binding protein CysA | 1.55 | 2.96 | 1.88 | 2.17 | 2.28 |
| RS12640 | 2423 | *cysW* |  | Y | sulfate/thiosulfate ABC transporter permease CysW | 2.14 | 4.43 | 2.52 | 2.81 | 2.72 |
| RS12645 | 2424 | *cysT* |  | Y | sulfate/thiosulfate ABC transporter permease CysT | 2.67 | 5.29 | 2.8 | 2.93 | 3.42 |
| RS12650 | 2425 | *cysP* |  | Y | thiosulfate/sulfate ABC transporter substrate-binding protein CysP | 2.87 | 5.43 | 2.95 | 3.37 | 3.93 |
| RS13135 | 2518 | *ndk* |  |  | nucleoside-diphosphate kinase | 0.57 | 1.41 | 1.11 | 1.04 | 1.1 |
| RS13540 | 2592 | *clpB* |  |  | ATP-dependent chaperone ClpB | 1.24 | 1.21 | 0.58 | 1.63 | 1.97 |
| RS13895 | 2669 | *stpA* |  |  | DNA-binding protein StpA | 1.61 | 2.91 | 1.37 | 1.46 | 2.02 |
| RS13950 | 2681 | *pseudo* |  |  | MFS transporter | 2.58 | 3.91 | 3.42 | 2.5 | 3.25 |
| RS14305 | 2749 | *ygbE* |  |  | DUF3561 family protein | 1.15 | 1.08 | 1.25 | 1.08 | 0.27 |
| RS14310 | 2750 | *cysC* |  | Y | adenylyl-sulfate kinase | 0.92 | 3.11 | 1.54 | 1.64 | 1.82 |
| RS14315 | 2751 | *cysN* |  | Y | sulfate adenylyltransferase subunit CysN | 1.18 | 3 | 1.62 | 2.04 | 1.83 |
| RS14320 | 2752 | *cysD* |  | Y | sulfate adenylyltransferase subunit CysD | 1.74 | 4.2 | 2.39 | 2.54 | 2.71 |
| RS14370 | 2762 | *cysH* |  | Y | phosphoadenosine phosphosulfate reductase | 0.73 | 1.27 | 1.14 | 1.44 | 1.09 |
| RS14375 | 2763 | *cysI* |  | Y | assimilatory sulfite reductase (NADPH) hemoprotein subunit | 1.19 | 3.13 | 1.53 | 1.79 | 1.97 |
| RS14380 | 2764 | *cysJ* |  | Y | NADPH-dependent assimilatory sulfite reductase flavoprotein subunit | 2.03 | 5.45 | 2.18 | 2.45 | 3.38 |
| RS14545 | 2796 | *sdaC* |  |  | HAAAP family serine/threonine permease SdaC | 1.37 | 2.35 | 1.69 | 1.37 | 1.86 |
| RS14550 | 2797 | *sdaB* |  |  | L-serine ammonia-lyase II | 1.25 | 2.44 | 1.6 | 1.5 | 1.92 |
| RS15025 | 2889 | *idi* |  |  | isopentenyl-diphosphate Delta-isomerase | 2.47 | 2.33 | 3.42 | 3.29 | 3.06 |
| RS15300 | 2946 | *rsmE* |  |  | 16S rRNA (uracil(1498)-N(3))-methyltransferase | 1.25 | 1.57 | 1.83 | 1.86 | 1.87 |
| RS15305 | 2947 | *gshB* | Y | Y | glutathione synthase | 1.33 | 1.65 | 1.91 | 2.21 | 1.92 |
| RS15435 | 2972 | *pppA* |  |  | prepilin peptidase PppA | 2.66 | 2.12 | 3.25 | 2.66 | 2.12 |
| RS15440 | 4466 | *sslE* |  |  | lipoprotein metalloprotease SslE | 3.12 | 3 | 3.38 | 3.12 | 3.42 |
| RS15715 | 3028 | *mdaB* | Y |  | NADPH:quinone oxidoreductase MdaB | 1.61 | 1.58 | 2.52 | 2.63 | 2.67 |
| RS15830 | 3050 | *yqiJ* |  |  | DUF1449 family protein | 2.42 | 1.74 | 2.32 | 2.42 | 2.22 |
| RS16380 | 3160 | *yhbW* | Y |  | luciferase-like monooxygenase | 3.03 | 3.36 | 3.74 | 4.05 | 4.22 |
| RS16560 | 3195 | *mlaF* | Y |  | phospholipid ABC transporter ATP-binding protein MlaF | 0.86 | 1.18 | 1.18 | 1.07 | 1.14 |
| RS16785 | 3238 | *yhcN* |  |  | peroxide/acid stress response protein YhcN | 5.38 | 5.04 | 5.42 | 3.23 | 3.78 |
| RS19010 | 3661 | *nlpA* |  |  | lipoprotein NlpA | 1.32 | 1.98 | 1.52 | 2.13 | 0.9 |
| RS19025 | 3662 | *nepI* |  |  | purine ribonucleoside efflux pump NepI | 1.58 | 2.46 | 2.09 | 1.39 | 1.58 |
| RS19170 | 3688 | *yidQ* |  |  | YceK/YidQ family lipoprotein | 1.74 | 1.83 | 1.99 | 1.99 | 1.48 |
| RS20325 | 3917 | *sbp* |  | Y | sulfate/thiosulfate ABC transporter substrate-binding protein Sbp | 2.12 | 3.57 | 3.12 | 3.43 | 2.88 |
| RS20475 | 3945 | *gldA* |  |  | bifunctional L-1,2-propanediol dehydrogenase/glycerol dehydrogenase | 1.38 | 1.58 | 1.38 | 1.58 | 1.58 |
| RS21085 | 4060 | *yjcB* |  |  | YjcB family protein | 1.91 | 1.58 | 2.39 | 1.46 | 1.38 |
| RS22530 | 4326 | *iraD* |  |  | anti-adapter protein IraD | 2 | 1.95 | 2.75 | 2.95 | 1 |
| RS22900 | 4396 | *robA* | Y |  | MDR efflux pump AcrAB transcriptional activator RobA | 1.18 | 1.72 | 1.36 | 1.13 | 1.31 |
| RS24030 |  | *wbbL* |  |  | N-acetylglucosaminyl-diphospho-decaprenol L-rhamnosyltransferase WbbL | 0.77 | 1.4 | 1.01 | 1.31 | 1.51 |
| RS25840 |  | *idlP* |  |  | iraD leader peptide IdlP | 2.35 | 2.61 | 2.98 | 3.15 | 1.58 |

**Table S3**: Transcriptional stress response of *E. coli ∆tolC* under aerobic conditions following 1 h exposure to sub MIC concentrations of MTZ in combination with the MTZ potentiators. Data is expressed as a fold change in gene expression (log_2_(rpkm)) caused by the co-incubation of low dose MTZ (0.1 mg/mL) with **BDM76441** (6 µM) or **BDM73031** (150 µM), compared to low dose MTZ alone (which practically has no stress response signature). In addition, the stress response induced by high dose (2 mg/mL) is also compared to low dose MTZ. Overall, booster-MTZ combinations induce genes of the SOS stress response (LexA regulon), similar to that seen for high dose MTZ. The selected genes are those found to be more than 2 fold dysregulated (log_2_ =1) in both MTZ potentiator combination studies.

| **[---Change in Log(2)_RPKM---]** | | | | | | | | |
| --- | --- | --- | --- | --- | --- | --- | --- | --- |
| **locus_tag BW25113_** | **old_locus_tag BW25113_** | **Gene Names** | **LexA regulon** | **Gene Product Description** | **Response to 6 µM BDM76441 with 0.1 mg/mL MTZ vs 0.1 mg/mL MTZ** | **Response to 150 µM BDM73031 with 0.1 mg/mL MTZ vs 0.1 mg/mL MTZ** | **Response to 2 mg/mL MTZ vs 0.1 mg/mL MTZ** |  |
| RS00300 | 0059 | *rapA* |  | RNA polymerase-associated protein RapA | 1.42 | 1.21 | 1.35 |  |
| RS00305 | 0060 | *polB* | Y | DNA polymerase II | 2.28 | 2.50 | 1.34 |  |
| RS01170 | 0231 | *dinB* | Y | DNA polymerase IV | 2.70 | 2.89 | 1.68 |  |
| RS01175 | 0232 | *yafN* | Y | Type I toxin anti-toxin system antitoxin YafN | 1.90 | 1.54 | 1.13 |  |
| RS01180 | 0233 | *yafO* | Y | Type II toxin anti-toxin system mRNA interferase toxin YafO | 2.08 | 1.84 | 1.23 |  |
| RS01185 | 0234 | *yafP* | Y | GNAT family N-acetyltransferase | 2.12 | 1.93 | 1.00 |  |
| RS04045 | 0779 | *uvrB* | Y | Excinuclease ABC subunit B | 1.33 | 1.27 | 1.31 |  |
| RS04145 | 0798 | *ybiA* |  | N-glycosidase YbiA | 1.11 | 1.13 | 0.44 |  |
| RS04150 | 0799 | *dinG* | Y | ATP dependent DNA helicase DinG | 1.79 | 1.87 | 0.74 |  |
| RS04165 | 0802 | *ybiJ* |  | DUF1471 family protein YbiJ | 1.22 | 1.37 | 3.38 |  |
| RS04970 | 0958 | *sulA* | Y | Cell division inhibitor SulA | 3.68 | 3.76 | 2.46 |  |
| RS05530 | 1061 | *dinI* | Y | DNA damage inducible protein I | 3.42 | 3.51 | 1.88 |  |
| RS06160 | 1183 | *umuD* | Y | Translesion error prone DNA polymerase V auto proteolytic subunit | 3.35 | 3.77 | 2.11 |  |
| RS06165 | 1184 | *umuC* | Y | DNA polymerase V catalytic protein | 3.04 | 3.84 | 2.28 |  |
| RS08630 | 1649 | *nemR* |  | DNA binding transcriptional regulator NemR | 2.42 | 2.09 | 2.93 |  |
| RS08635 | 1650 | *nemA* |  | N-ethylmaleimide reductase | 2.30 | 2.23 | 3.39 |  |
| RS08680 | 1658 | *purR* |  | HTH-type transcriptional repressor PurR | 1.12 | 1.71 | 0.08 |  |
| RS09040 | 1728 | *ydjM* | Y | Metal dependent hydrolase | 2.91 | 2.75 | 1.72 |  |
| RS09110 | 1741 | *cho* | Y | Excinuclease Cho | 2.58 | 2.55 | 1.39 |  |
| RS09460 | 1808 | *yoaA* |  | ATP-dependent DNA helicase | 1.08 | 1.11 | 0.74 |  |
| RS09685 | 1847 | *yebF* | Y | Protein YebF | 1.74 | 1.63 | 1.16 |  |
| RS09690 | 1848 | *yebG* | Y | DNA damage inducible protein YebG | 2.98 | 3.35 | 1.70 |  |
| RS09755 | 1860 | *ruvB* | Y | Holliday junction branch migration DNA helicase RuvB | 1.45 | 1.44 | 1.07 |  |
| RS09760 | 1861 | *ruvA* | Y | Holliday junction branch migration protein RuvA | 1.63 | 1.71 | 1.12 |  |
| RS10495 | 2008 | *yeeA* |  | FUSC family protein | 1.47 | 1.37 | 0.34 |  |
| RS10500 | 2009 | *sbmC* | Y | DNA gyrase inhibitor SbmC | 2.08 | 2.01 | 0.62 |  |
| RS12665 | 2428 | *murQ* |  | N-acetyl muramic acid 6phosphate etherase | 1.51 | 1.25 | 1.64 |  |
| RS12670 | 2429 | *murP* |  | PTS N-acetyl muramic acid transporter subunit IIBC | 1.38 | 1.15 | 1.75 |  |
| RS12675 | 2430 | *pbp4b* |  | Penicillin binding protein PBP4B | 1.36 | 1.09 | 1.87 |  |
| RS13655 | 2616 | *recN* | Y | DNA repair protein RecN | 4.24 | 4.30 | 2.44 |  |
| RS14045 | 2698 | *recX* | Y | Recombination regulator RecX | 2.29 | 2.78 | 2.00 |  |
| RS14050 | 2699 | *recA* | Y | Recombinase RecA | 2.59 | 2.75 | 1.92 |  |
| RS18130 | 4613 | *dinQ* | Y | Hypothetical protein | 1.72 | 2.42 | 0.81 |  |
| RS18925 | 3645 | *dinD* | Y | DNA damage inducible protein D | 2.70 | 3.11 | 1.46 |  |
| RS19090 | 4618 | *tisB* | Y | Type I toxin anti-toxin system toxin TisB | 3.18 | 3.78 | 2.31 |  |
| RS19800 | 3813 | *uvrD* | Y | DNA helicase II | 1.48 | 1.43 | 1.08 |  |
| RS19895 | 3832 | *rmuC* |  | DNA recombination protein RmuC | 1.77 | 2.00 | 0.72 |  |
| RS20995 | 4043 | *lexA* | Y | Transcriptional repressor LexA | 2.74 | 2.72 | 1.44 |  |
| RS21000 | 4044 | *dinF* | Y | MATE family efflux transporter DinF | 2.88 | 2.89 | 1.47 |  |
| RS21075 | 4058 | *uvrA* | Y | Excinuclease ABC subunit UvrA | 1.72 | 1.88 | 0.80 |  |
| RS22630 | 4347 | *symE* | Y | Endoribonuclease SymE | 1.91 | 2.09 | 1.09 |  |

**Table S4:** DFT-computed hyperfine couplings constants for MTZ radical anion.

|  | A-tensor eigenvalues | | |  |
| --- | --- | --- | --- | --- |
| Center | A_min_ | A_mid_ | A_max_ | A_iso_ |
| 2N | -1.7 | -1.8 | -4.3 | -2.6 |
| 4N | 0.9 | 1.2 | 7.3 | 3.1 |
| 7N | 7.4 | 7.5 | 67.6 | 27.5 |
| 1C | 19.1 | 20.0 | 67.4 | 35.5 |
| 3C | 6.7 | 7.6 | 30.1 | 14.8 |
| 10C | -4.4 | -4.7 | -4.7 | -4.5 |
| 14C | -0.9 | -1.7 | -2.0 | -1.5 |
| 17C | 5.8 | 6.0 | 7.3 | 6.4 |
| 6H | -9.3 | -19.2 | -23.3 | -17.3 |
| 11H | -0.4 | -0.7 | 2.2 | 0.4 |
| 12H | 8.7 | 8.9 | 11.3 | 9.7 |
| 13H | 11.6 | 11.6 | 14.1 | 12.4 |
| 15H | -0.3 | 1.0 | 6.5 | 2.4 |
| 16H | -0.1 | -0.9 | 2.2 | 0.4 |
| 18H | -1.5 | -2.3 | 3.4 | -0.2 |
| 19H | -0.1 | -0.3 | 2.1 | 0.6 |
| 21H | -0.5 | -1.0 | 1.6 | 0.1 |

**Table S5**: DFT-computed hyperfine couplings constants for NFZ radical anion.

|  | A-tensor eigenvalues | | | |
| --- | --- | --- | --- | --- |
| Center | A_1_ | A_2_ | A_3_ | A_iso_ |
| 8N | 2.6 | 2.7 | 40.8 | 15.4 |
| 13N | 3.8 | 4.1 | 31.2 | 13.0 |
| 14N | -0.9 | -1.2 | 3.9 | 0.6 |
| 18N | 0.1 | 0.1 | 0.2 | 0.1 |
| 1C | 14.9 | 15.4 | 47.7 | 26.0 |
| 2C | -14.9 | -15.6 | -36.2 | -22.2 |
| 3C | 14.2 | 15.8 | 59.7 | 29.9 |
| 5C | -16.9 | -21.0 | -30.2 | -22.7 |
| 11C | -14.0 | -17.1 | -29.9 | -20.4 |
| 16C | -0.5 | 1.1 | -1.5 | -0.3 |
| 6H | -6.5 | -13.4 | -15.8 | -11.9 |
| 7H | 4.4 | 4.6 | 10.1 | 6.3 |
| 12H | 1.2 | 2.4 | 6.6 | 3.4 |
| 15H | 0.3 | -5.1 | -5.5 | -3.5 |
| 19H | -0.9 | -1.1 | 2.4 | 0.1 |
| 20H | -0.6 | -0.6 | 0.8 | -0.1 |

**Table S6**: List of primers used in this study

| **Primers** | **Sequence (5’-3’)** | **Notes (used for)** |
| --- | --- | --- |
| **RH698** | CTTATTTGTGGCCATCGCTCCATTCGCCATTTCACTGATGAACCCGTGTAGGCTGGAGCTGCTTC | *nfsA* disruption with KmR cassette |
| **RH699** | GCCTTTATCCAGCGGTTGATAGCTGTTTTCATGCACCAAAATGGACATATGAATATCCTCCTTAG | *nfsA* interruption with KmR cassette |
| **RH704** | TGCCAGCAAAAAACTTACCCCGGAACAGGCCGAGCAGATCAAAACGTGTAGGCTGGAGCTGCTTC | *nfsB* interruption with KmR cassette |
| **RH705** | TAGCGTTAAAATCTTCAACGCTGTGATGACCTACCGGAACAACCACATATGAATATCCTCCTTAG | *nfsB* interruption with KmR cassette |
| **RH710** | AATCCATATGGTTAATCAGAAGAAAGATCGCCTGCTTAACGAGTAGTGTAGGCTGGAGCTGCTTC | *marR* interruption with KmR cassette |
| **RH711** | CCGTCAGGTTTTTTGTTAATTCTTGGTGCAGGTCCTGGCCAACTAATTGATCATATGAATATCCTCCTTAG | *marR* interruption with KmR cassette |
| **RH716** | GATCGAGGACAACCTGGAATCGCCACTGTCACTGGAGAAAGTGTCGTGTAGGCTGGAGCTGCTTC | *marA* interruption with KmR cassette |
| **RH717** | TATTGGTCATCCGGTATTTATGCGGCGGAACATCAAAGTAATTTTCATATGAATATCCTCCTTAG | *marA* interruption with KmR cassette |
| **RH722** | AATAGCAGCTGCGCTTATTCTCTTTTCCGCGCAGGGCGTTGCGGAGTGTAGGCTGGAGCTGCTTC | *marB* interruption with KmR cassette |
| **RH723** | AATAGGGCACGCCGAGCGCATCCGACTTATCACTGCCAGTACCCACATATGAATATCCTCCTTAG | *marB* interruption with KmR cassette |
| **RH770** | TATTGACCAGCCGCTTAACATTGATGTAGTCGCAAAAAAATCAGGGTGTAGGCTGGAGCTGCTTC | *soxS* interruption with KmR cassette |
| **RH771** | AAACGCGGGAGAAGGTCTGCTGCGAGACATAACCCAGGTCCATTGCATATGAATATCCTCCTTAG | *soxS* interruption with KmR cassette |
| **RH776** | TCTGGATCAGCCCCTGTCGCTCGACAATGTAGCGGCGAAAGCAGGGTGTAGGCTGGAGCTGCTTC | *rob* interruption with KmR cassette |
| **RH777** | CTCCCGCTTTGGCATCTTCTGCCGGGTAGTATCGCTCAATATCCTCATATGAATATCCTCCTTAG | *rob* interruption with KmR cassette |
| **RH980** | GGTCTAGAAAGAGAAAAAGATAATGACGCCAACCATTGAACTTATTTGTGGCC | *nfsA* cloning into pBAD30 |
| **RH840** | GGGTCGACTTAGCGCGTCGCCCAACCCTG | *nfsA* cloning into pBAD30 and pET28a |
| **RH839** | GGCATATGACGCCAACCATTGAACTTATTTGTGGCC | *nfsA* cloning into pET28a |
| **RH842** | GGCATATGAAAAGTACCAGCGATCTGTTCAATGAAATTATTCCATTGGGTCGCTTAATCCACATG | *marR* cloning into pET15b |
| **RH843** | GGCTCGAGTTACGGCAGGACTTTCTTAAGCAAATACTCAAGTGTTGCC | *marR* cloning into pET15b |
| **RH968** | GGTCTAGATGGAGTCTTTATGGATATCATTTCTGTC | *nfsB* cloning into pBAD30 |
| **RH962** | GGGTCGACTTACACTTCGGTTAAGGTGA | *nfsB* cloning into pBAD30 |
| **RH1125** | tggatcgcctggtcagcaaaggctgggtg | to generate *marR*-C80S |
| **RH1126** | cacccagcctttgctgaccaggcgatcca | to generate *marR*-C80S |
| **RH1127** | CGACCTGGGAGCACTGACCCGTATGCTGGATCGCCTGGTCAGCAAGTGTAGGCTGGAGCTGCTTC | *marR* disruption by KmR cassette |
| **RH1128** | ACGCCGCGCTTGTCATTCGGGTTCGGCAACCTTTCCACCCAGCCTCATATGAATATCCTCCTTAG | *marR* disruption by KmR cassette |
| **RH1129** | AGCGATCTGTTCAATGAAAT | *marR*-C80S amplification |
| **RH1130** | TCTTAAGCAAATACTCAAGT | *marR*-C80S amplification |
| **RH957** | GTCAATTCATTCATTTGACT | *marO* amplification |
